# Single-Cell and Spatial Transcriptomics Reveal That *TXNIP* and *BIRC3* Contribute to Human Prostate Tumor Progression

**DOI:** 10.3390/cells15070647

**Published:** 2026-04-02

**Authors:** Seyed Taleb Hosseini, Hossein Azizi, Thomas Skutella

**Affiliations:** 1 Immunogenetics Research Center, Mazandaran University of Medical Sciences, Sari 48471-91971, Iran; seyedtalebhouseini@gmail.com; 2 Department of Biology, QaS.C., Islamic Azad University, Qaemshahr 61964-47651, Iran; 3 Department of Stem Cells and Cancer, College of Biotechnology, Amol University of Special Modern Technologies, Amol 46166-86767, Iran; 4 Institute for Anatomy and Cell Biology, Medical Faculty, University of Heidelberg, Im Neuenheimer Feld 307, 69120 Heidelberg, Germany

**Keywords:** prostate cancer, precision oncology, biomarker discovery, tumor heterogeneity, single-cell RNA seq, spatial transcriptomics

## Abstract

Prostate cancer is one of the most prevalent malignancies among men and remains a major clinical challenge due to the complex tumor microenvironment. Understanding gene expression dynamics at both cellular and spatial levels is essential for improving therapeutic strategies. In this study, we performed an integrated multi-omics analysis using single-cell RNA sequencing and spatial transcriptomics. scRNA-seq data from 15 prostate samples, including 8 normal and 7 tumor tissues, were analyzed to characterize distinct cellular populations. Spatial transcriptomic profiling was conducted on three FFPE prostate tissue sections, including adjacent normal tissue, acinar cell carcinoma, and invasive adenocarcinoma, using the standard 10x Genomics Visium FFPE platform (55 µm capture spots). Single-cell analysis revealed heterogeneity among epithelial, stromal, and immune cell populations, highlighting complex signaling networks in which myeloid cells may contribute to tumor progression through immune suppression and epithelial adaptability. Spatial transcriptomic analysis further identified region-specific expression patterns and spatially restricted tumor niches, including the regional establishment of *TXNIP* and *BIRC3* as genes associated with metabolic stress and inflammatory survival pathways. The spatial colocalization of *BIRC3* with tumor vasculature in invasive carcinoma tissue suggests a novel interaction. Our discoveries using an integrated single-cell and spatial transcriptomic approach reveal a high-resolution molecular map of prostate cancer with spatial features that may provide further therapeutic investigation.

## 1. Introduction

Prostate cancer is one of the most common malignancies affecting men worldwide and represents a major public health concern due to its high incidence and variable clinical outcomes [1]. According to global cancer statistics, prostate cancer is the second most frequently diagnosed cancer in men, with more than 1.4 million new cases reported annually [2]. The incidence of prostate cancer is highest in North America, Western Europe, and Oceania, whereas mortality rates are disproportionately higher in Africa and parts of Asia, reflecting disparities in early detection and access to treatment [3]. Age is the strongest risk factor for prostate cancer, with most cases diagnosed in men older than 65 years, highlighting the role of aging-related biological changes in disease development [4]. Environmental and lifestyle factors, including high-fat diets, obesity, physical inactivity, and chronic inflammation, have been linked to an increased risk of prostate cancer initiation and progression [5]. In addition to environmental influences, genetic susceptibility plays a significant role in prostate cancer, as men with a positive family history exhibit a substantially higher risk of developing the disease [6]. Germline and somatic alterations in key genes such as *BRCA1*, *BRCA2*, *PTEN*, and *TP53* are associated with aggressive tumor behavior, poor prognosis, and resistance to conventional therapies [7]. Tumor progression in prostate cancer is driven by the accumulation of genetic and epigenetic abnormalities that promote uncontrolled cell proliferation, evasion of apoptosis, and metastatic potential [7]. Recent advances in genomic technologies have greatly improved our understanding of prostate cancer heterogeneity at the molecular level [8]. Single-cell RNA sequencing has emerged as a powerful tool to dissect cellular diversity within prostate tumors and to identify rare cell populations associated with disease progression and drug resistance [9]. Spatial transcriptomics further complements single-cell approaches by preserving tissue architecture, enabling the investigation of spatial interactions between tumor cells and the surrounding microenvironment [10]. The integration of single-cell and spatial transcriptomic data facilitates the discovery of novel diagnostic and prognostic biomarkers in prostate cancer [11]. Ultimately, these high-resolution genomic approaches support the development of precision medicine strategies tailored to the molecular characteristics of individual prostate tumors [12]. *TXNIP* (thioredoxin-interacting protein) is a regulator of cellular redox balance and metabolic stress responses and has been implicated in metabolic reprogramming and oxidative stress signaling in several cancers, including prostate cancer [13]. *BIRC3* (baculoviral IAP repeat containing 3), a member of the inhibitor of apoptosis (IAP) family, is involved in inflammatory signaling pathways and the regulation of cell survival through NF-κB-associated mechanisms [14]. Emerging evidence suggests that dysregulation of these pathways may contribute to tumor progression and microenvironmental adaptation in prostate cancer [15].

In this research, we conducted an integrated multi-omics investigation of prostate cancer (PCa) utilizing single-cell and spatial transcriptomic data. We analyzed single-cell RNA-seq data from 15 PCa samples, including 8 normal and 7 tumor tissues. Spatial transcriptomic profiling was performed on three formalin-fixed paraffin-embedded (FFPE) PCa tissue sections, including (1) Adjacent Normal Section with IF Staining (FFPE), (2) Acinar Cell Carcinoma (FFPE) and (3) Invasive Adenocarcinoma (FFPE), which were obtained from the standard 10x Genomics Visium FFPE platform, (10x Genomics, Pleasanton, CA, USA) (55 µm capture spots). Our study demonstrates the cellular heterogeneity of the prostate tumor microenvironment, maps intercellular communication networks, and identifies cell-type and spatially resolved gene expression patterns associated with cancer progression. These findings provide a high-resolution molecular atlas that may uncover context-dependent biomarkers and therapeutic targets for prostate cancer.

## 2. Materials and Methods

### 2.1. Collecting RNA-Seq Datasets (Single-Cell RNASeq and Spatial Transcriptomics)

**Figure 1:**
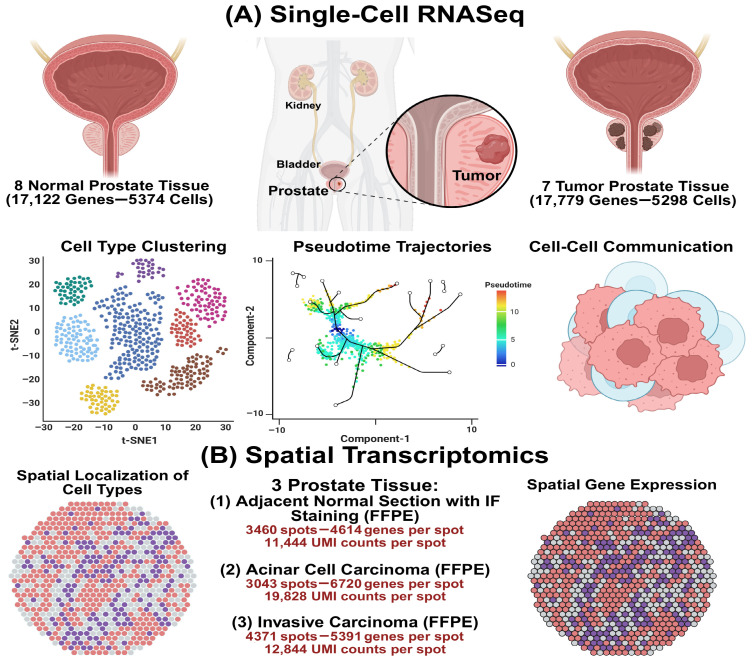
Integrated workflow for single-cell and spatial transcriptomic profiling in prostate cancer. (**A**) Single-cell RNA-seq data from 15 human prostate samples (8 normal and 7 tumors; 10,672 cells and 34,901 genes) were processed for quality control, normalization, clustering, cell type annotation, pseudotime trajectory analysis, and CellChat-based cell-cell communication inference to characterize cellular diversity and intercellular signaling networks. (**B**) Spatial transcriptomic data obtained from publicly available 10x Genomics standard Visium FFPE prostate tissue datasets, including adjacent normal prostate tissue, acinar cell carcinoma, and invasive carcinoma, were analyzed at spot-level resolution to map spatial gene expression patterns and tissue heterogeneity.

The methodological pipeline and analytical framework of this research are indicated in Figure 1. With the objective of identifying gene expression profiles associated with prostate cancer (PCa) using both single-cell RNA-Seq and spatial transcriptomics, we performed an extensive search across the Sequence Read Archive (SRA, https://www.ncbi.nlm.nih.gov/sra) [16], Gene Expression Omnibus (GEO, https://www.ncbi.nlm.nih.gov/geo/) [17], and PubMed (https://pubmed.ncbi.nlm.nih.gov/). The search parameters were defined using terms such as “Prostate Cancer”, “Prostate Adenocarcinoma”, “Castration-Resistant Prostate Cancer”, “bulk RNASeq”, “single cell RNASeq”, “gene expression profiling”, “spatial heterogeneity”, “Visium FFPE gene expression” and “spatial transcriptomics analysis”. The 10x Genomics database (https://www.10xgenomics.com/) was utilized as the primary source for acquiring datasets related to spatial transcriptomics. Our database inquiry followed rigorous standards specific to spatial transcriptomics, emphasizing the use of Visium FFPE spatial gene expression technology or suitable 10x-compatible spatial profiling methods. The search criteria integrated terms like “Spatial transcriptomics”, “Visium FFPE” and “Spatial gene expression”, alongside parameters for clinical metadata (including tissue source, species, and sequencing depth) and resource formats (such as raw FASTQ files and processed count matrices). Relevant files, typically encompassing raw sequencing reads, spatial barcode information, and histological imaging data, were retrieved to obtain appropriate datasets. We assessed the comparability of these datasets by integrating their metadata with our research goals to ensure data precision and scientific significance. The study included single-cell RNA-Seq and spatial transcriptomics datasets, providing gene expression levels for various cell types within prostate tumor tissues and healthy prostate tissues. Single-cell and spatial transcriptomics datasets from animal models, such as rat and mouse studies and involving various cell types treated with different drugs or antibodies, as well as systematic reviews, were excluded from the analysis.

### 2.2. Characterization of Datasets: Single-Cell RNA-Seq and Spatial Transcriptomics

We obtained original expression RNA-Seq datasets, including single-cell RNA sequencing (scRNA-seq) and spatial transcriptomics (ST). The collection encompasses 18 samples, with 10,672 cells and 34,901 genes from the single-cell dataset and 10,874 spots from the 10x Genomics spatial datasets. Specifically, single-cell RNA sequencing (scRNA-seq), which resolves transcriptomic profiles at the cellular level, utilized the high-quality GSE176031 dataset from the Gene Expression Omnibus (GEO) [https://www.ncbi.nlm.nih.gov/geo/query/acc.cgi?acc=GSE176031, accessed on 27 March 2026] [18]. This data, sequenced using the Illumina HiSeq 2000 platform (Illumina, Inc., San Diego, CA, USA), includes 15 total human prostate cancer (PCa) samples, including 8 normal prostate tissue samples (17,122 genes, 5374 cells) and 7 prostate tumor samples (17,779 genes, 5298 cells), providing cellular resolution for expression profiling across different conditions. For spatial transcriptomics, integrating gene expression with spatial coordinates, three datasets were directly sourced from the 10x Genomics public database, utilizing the standard Visium FFPE spatial gene expression technology and sequenced on the Illumina NovaSeq platform (Illumina, Inc., San Diego, CA, USA). These spatial datasets include Human Prostate Cancer, Adjacent Normal Section with IF Staining (FFPE) [https://www.10xgenomics.com/datasets/human-prostate-cancer-adjacent-normal-section-with-if-staining-ffpe-1-standard, accessed on 27 March 2026] from stage II adenocarcinoma tissue, featuring 3460 spots with a median of 4614 genes per spot and 11,444 UMI counts per spot; aggressive stage IV Human Prostate Cancer, Acinar Cell Carcinoma (FFPE) [https://www.10xgenomics.com/datasets/human-prostate-cancer-acinar-cell-carcinoma-ffpe-1-standard, accessed on 27 March 2026], comprising 3043 spots, a median of 6720 genes per spot, and 19,828 UMI counts per spot; and the stage III Human Prostate Cancer, Adenocarcinoma with Invasive Carcinoma (FFPE) [https://www.10xgenomics.com/datasets/human-prostate-cancer-adenocarcinoma-with-invasive-carcinoma-ffpe-1-standard-1-3-0, accessed on 27 March 2026], which included 4371 spots, yielding a median of 5391 genes per spot and 12,844 UMI counts per spot. Spatial transcriptomics data were obtained from the 10x Genomics public repository and generated using the standard 10x Genomics Visium FFPE platform. The platform provides spot-level spatial resolution with capture spots of approximately 55 µm in diameter. These integrated datasets provide a robust multi-omics framework, combining cellular-level and spatially resolved context for comprehensive investigation of PCa progression.

### 2.3. Quality Control and Data Integration in Single-Cell Transcriptomics Analysis

The Seurat package (v5.4.0) [19] was employed for the analysis of genomics scRNA-seq data (10,672 cells, 34,901 genes) within the R programming language (v 4.3). Quality control (QC) was performed on the input matrix, and low-quality cells were removed based on specific criteria to establish a high-quality expression matrix: (1) to initialize a Seurat object, cells were required to express more than 200 distinct genes, and genes had to be detected in at least three different cells; (2) regarding total diversity, only cells with gene expression levels between 200 and 4500 for normal prostate objects and 4500 for prostate cancer objects were evaluated; (3) the “PercentageFeatureSet” function was used to calculate the proportion of genes associated with ribosomal or mitochondrial functions within each cell. The scRNA-seq data were normalized using the “LogNormalize” method via the “NormalizeData” function. Following quality assessment, the top 2000 highly variable genes were identified and visualized using the “FindVariableFeatures” and “VariableFeaturePlot” functions. The “SelectIntegrationFeatures”, “FindIntegrationAnchors”, and “IntegrateData” methods were utilized to integrate all Seurat objects (normal and prostate tumor) through the CCA algorithm. Subsequently, the expression data for every gene were adjusted and centered using the “ScaleData” function, ensuring a variance of 1 and a mean expression of 0 for each cell. Principal component analysis (PCA) was performed on the 2000 highly variable genes using the “RunPCA” function in Seurat [19]. We conducted a comprehensive cellular clustering analysis based on the first 20 principal components. The functions “VizDimLoadings”, “DimPlot”, and “DimHeatmap” were used to visualize gene expression contributions to the PCAs. Following this, clusters were identified using “JackStraw (num.replicate = 100)”, “ScoreJackStraw (dims = 1:20)”, “JackStrawPlot”, “ElbowPlot”, “FindNeighbors (dims = 1:20)”, and “FindClusters” within Seurat [19], with the resolution parameter set to 0.5. Additionally, the T-distributed Stochastic Neighbor Embedding (TSNE) technique via the “RunTSNE” function was applied for dimensionality reduction and cluster discovery. The “FindAllMarkers” function was used to determine the log2 fold change and false discovery rates (Adj.Pvalue) to identify differentially expressed genes (DEGs) for each cluster. DEGs with the threshold of logfc = 1 and min.pct = 0.25 were designated as marker genes. Finally, the SingleR (v2.12.0) [20], celldex (1.20.0) [20], and SingleCellExperiment (v1.32.0) [21] R packages (v4.5) were used to computationally categorize cell types using the “HumanPrimaryCellAtlasData” function. The ClusterProfiler package (v4.18.4) [22] in the R programming language was applied to identify GO and KEGG functional characteristics of the marker genes in critical cell populations.

### 2.4. Single-Cell Pseudotime Trajectories Analysis

The R package Monocle2 (v2.30.0) [23] was used to perform single-cell trajectory analysis to determine changes in cellular states. RDS data containing cell types were imported into the R environment. The “newCellDataSet” function was utilized to create a new object with “expressionFamily = negbinomial.size” and “lowerDetectionLimit = 0.5”. Dimensionality reduction was performed using the “reduceDimension” function with “reduction_method = ‘DDRTree’” and “max_components = 2”. Cell lineage trajectories were then defined through pseudotime and clustering using the default parameters of Monocle2 [23]. The root state was assigned using Monocle2’s default ordering procedure based on the learned DDRTree manifold. We also inspected the distribution of tumor and normal cells along the trajectory to confirm that pseudotime reflected continuous transcriptional transitions rather than simple sample separation. The overall trajectory topology remained stable under minor parameter variations within the Monocle2 framework. Findings were visualized using the “plot_cell_trajectory” function. Furthermore, dynamic shifts in pseudotime-dependent gene expression were visualized using the “plot_genes_in_pseudotime” function.

### 2.5. Investigation of Cell-to-Cell Communication

Intercellular communication networks were comprehensively modeled from scRNA-seq data using the CellChat package (v2.1.2) [24]. CellChat (https://github.com/sqjin/CellChat, accessed on 27 March 2026) predicts essential signaling inputs and outputs for cells and identifies how specific signaling pathways are connected based on human ligand-receptor interactions and pattern detection algorithms [24]. Potential interaction networks were discovered for each prostate cancer group. CellChat was built upon a database of secreted signaling, ECM-receptor, and cell-cell contact interactions. Following established protocols, we used R to load RDS files containing normalized counts and cell types from Seurat [19] into CellChat (v2.1.2) [24]. Standard preprocessing functions, including “identifyOverExpressedGenes” and “identifyOverExpressedInteractions”, were applied. Key functions such as “aggregateNet”, “computeCommunProb”, and “computeCommunProbPathway” were used for the core analysis using comparable parameters. Communication networks were visualized within the R programming language.

### 2.6. Integrative Analysis of Gene Biomarkers: ROC, Survival and TCGA Expression Profiles

To evaluate the potential of selected genes as biomarkers, ROC curves were generated using the pROC [25] package (v1.19.0.1) and “roc” function to compare prostate tumor and normal samples and calculate AUC values. Results were visualized to highlight discriminative genes. AUC values of 0.7–0.8 were considered acceptable, 0.8–0.9 good, and 0.9–1 excellent. Subsequently, survival analysis was performed using the survival (v3.8-6) [26] and survminer (v0.5.2) [27] packages. Kaplan-Meier curves were created using the “Surv” and “survfit” functions, stratifying patients into high and low expression groups based on median expression, with hazard ratios and *p*-values calculated to assess prognostic significance. Finally, gene expression levels in tumor and normal tissues were examined using TCGA-PRAD (prostate adenocarcinoma) data obtained via the TCGAbiolinks [28] package (v2.38.0). Differences were visualized to validate the relevance of selected genes and provide an overview of their diagnostic and prognostic potential in prostate cancer.

### 2.7. Decoding Tissue Architecture Using Spatial Transcriptomics and Cell Deconvolution

In this deconvolution, the GraphST (v1.1) [29] package in the Python (v3.8) programming language was specifically used to infer the spatial distribution and relative abundance of cell types across visium capture spots by integrating scRNA-seq reference data with spatial transcriptomics profiles. These included GraphST for graph-based spatial analysis [29], OS for file management, Torch (v2.11) for deep learning assistance [30], Skmisc (v0.5.2)and Scikit-learn (v1.8.0) for statistical and metric calculations [31], OT for optimal transportation analysis [32], Matplotlib (v3.10.0) and its Pyplot submodule for visualization [33], Pandas (v3.0.1) [34] and NumPy (1.26.0) [35] for data manipulation, and Scanpy (v1.9.8) [36] and AnnData (v0.12.10) for processing spatial transcriptomics data. The scRNA-seq data, originally in RDS format, were converted to the AnnData-compatible h5ad format using the SeuratData (v0.2.1) [37] and SeuratDisk (v0.0.0.9015) [38] packages in the R programming language. Following this, the “scanpy.read_h5ad” function from Scanpy [36] was used to import the single-cell dataset into Python. The filtered feature-barcode matrix (filtered_feature_bc_matrix.h5) and related high-resolution images were loaded using the “scanpy.read_visium” function from the standard Visium FFPE platform. Using the “var_names_make_unique” function, unique variable names were assigned to both datasets to facilitate integration. To ensure analytical strength, the datasets underwent a thorough preparation process. We applied “sc.pp.normalize_total” to the spatial dataset (data_spatial) with a target sum of 10,000 counts per spot, followed by log-transformation using the “sc.pp.log1p” function. Highly variable genes (HVGs) were identified using the “sc.pp.highly_variable_genes” function, and the top 2000 genes were selected. To prepare spatial data for GraphST, we used the “GraphST.preprocess” function, constructed interactions with the “GraphST.construct_interaction” function, and added contrastive labels with the “GraphST.add_contrastive_label” function. The single-cell dataset (data_singlecell) underwent similar processing: the “sc.pp.normalize_total(target_sum = 1e4)” function for normalization and the “sc.pp.log1p” function for log-transformation were used. Negative expression values were set to zero via “data_singlecell.X[data_singlecell.X < 0] = 0”, and “np.nan_to_num” was used to replace incomplete or infinity values with zeros. We used the “sc.pp.filter_cells(min_genes = 1)” function and the “sc.pp.filter_genes(min_cells = 1)” function to filter the data. The dataset was subset based on 2000 HVGs using the “sc.pp.highly_variable_genes(n_top_genes = 2000)” function. The single-cell data were then aligned with spatial data using the “GraphST.preprocess” function. The “GraphST.preprocess.filter_with_overlap_gene” function harmonized the feature spaces of both datasets to preserve overlapping genes. To create a representation for modeling, the “GraphST.get_feature” function extracted spatial features. The GraphST model was initialized with 1200 epochs, a random seed of 50, and computation delegated to a CUDA GPU (torch.device(‘cuda:1’)) or CPU. The single-cell reference was used to determine cell proportions in each spot by setting the “deconvolution = True” function. By mapping characteristics onto the spatial context, the “model.train_map” function combined the datasets. The single-cell data were annotated with cell types according to a specified “frequencies” list. The “data_singlecell.obs[‘cell_type’]” column was updated with a “cell_type_list” created by repeating each type based on its frequency. The “GraphST.utils.project_cell_to_spot” function projected the annotations onto spatial spots, retaining 15% of the top features (“retain_percent = 0.15”). We used the “scanpy.pl.spatial” function to visualize the distribution of deconvoluted cell types. Key cell types for the prostate cancer group included epithelial cells, monocyte, T cells, endothelial cells, B cells, fibroblasts and neutrophils. By modeling the distribution according to spatial coordinates, we visualized the number of cells in various prostate tissue regions using the viridis color palettes.

### 2.8. Spatial Gene Expression Profiling

For gene expression analysis, 10x Genomics spatial transcriptome data were used to identify spatial expression patterns in prostate cancer. Spatial transcriptomics (ST) data were analyzed and visualized using the Seurat (v5.0.1) R package [19]. The “SCTransform” function was used for normalization, “ScaleData” for scaling, and “RunPCA”, “FindNeighbors”, “FindClusters”, and “RunUMAP” for dimensionality reduction and clustering. Integrated cell type interpretations were used to provide the scRNA-seq reference. Using the R package SpacexR (v.2.2.1) [39], the RCTD (Robust Cell Type Decomposition) framework was applied to analyze spatial gene expression patterns in the spatial transcriptomics data using scRNA-seq information as a reference. In this study, RCTD was used to characterize the spatial gene expression structure rather than to estimate cell type proportions, which were instead inferred using GraphST [29], as described above. For each standard Visium FFPE slice, matrices containing raw counts and coordinates were used to build the “spatialRNA” object in RCTD. The reference scRNA-seq and spatialRNA objects were processed using “create.RCTD”, and the RCTD algorithm was executed with the “doublet_mode = doublet” option via the “run.RCTD” function. The “SpatialFeaturePlot” function was used to identify the localization of specific genes.

### 2.9. Spatial Autocorrelation Analysis of Gene Expression and Cell Type Frequencies

Spatial autocorrelation analysis was conducted in the R programming language using Seurat [19], dplyr (v1.2.0) [40], and spdep (v1.4.2) [41]. Preprocessed datasets in H5Seurat format were imported using the “LoadH5Seurat” function, and normalized expression values for marker genes were extracted with the “GetAssayData” function. These profiles were merged with active cell type identities. For each tissue dataset, spatially matched cells were grouped by annotated types. Spatial autocorrelation within each cell type was evaluated using Moran’s I from spdep [41]. Neighbor relationships were defined through a 4-nearest-neighbor graph using the “knearneigh” and “knn2nb” functions, and weight matrices were generated utilizing the “nb2listw(style = ‘W’)” function. Moran’s I statistics and *p*-values were calculated using the “moran.test” function with “zero.policy = TRUE” to accommodate isolated points.

### 2.10. Statistical Analysis

All statistical analyses were performed using the R programming language (v4.3.2) and Python. Various packages were used for processing and visualization, including ClusterProfiler, Seurat, Celldex, SingleCellExperiment, SingleR, Monocle2, CellChat, SeuratDisk, SeuratData, SpacexR, dplyr, spdep, TCGAbiolinks, pROC, survival, survminer, Scanpy, Matplotlib, OS, Torch, Pandas, NumPy, Skmisc, Scikit-learn, OT, and GraphST. Cell frequencies measured by Xenium (https://www.10xgenomics.com/jp/platforms/xenium) were compared between groups using the Mann–Whitney test, with multiple comparisons controlled using the Benjamini, Krieger, and Yekutieli two-stage linear step-up procedure to manage the false discovery rate (FDR). We applied false discovery rate (FDR) correction and considered features significant at an FDR-adjusted *q*-value < 0.01.

## 3. Results

### 3.1. Exploring Cellular Diversity Through Single-Cell Transcriptome Profiling

We performed an scRNA-seq evaluation of 10,672 cells and 34,901 genes from 15 human prostate samples, comprising both normal adjacent and tumor tissues. Following the standardization and quality assessment of scRNA-seq data, cells of lower quality were eliminated (Supplementary Figure S1A). Finally, the top 2000 genes with significant variance variations were chosen for further examination (Supplementary Figure S1B). Next, we performed a dimension reduction assessment between samples by using the “RunPCA” function to lower the PCA dimensionality of the top 2000 highly variable genes (Supplementary Figure S1C). A large number of “important” PCs with low *p*-values were found. The top 20 principal components were displayed using the JackStrawPlot method, and then each PC’s variance was calculated and evaluated with the median dispersion (Supplementary Figure S1D). In general, the “important” PCs had a low *p*-value and fully aligned with the data of highly distinct genes. In contrast, the “ElbowPlot” function showed that although the flexible point appeared roughly at the 10th PC, the variance progressively diminished after 20 PC (Supplementary Figure S1E). In this step, we created a heatmap of the original PCs and highlighted their most significant genes with the goal of revealing how their DEGs differentiated from those of other PCs (Supplementary Figure S1F), and VizDimLoadings was used to visualize gene contributions to principal components (Supplementary Figure S1G). Finally, we identified the top 20 PCs for further TSNE evaluation.

### 3.2. Distinct Cellular Signatures and Abundance Patterns in Prostate Cancer Progression

**Figure 2:**
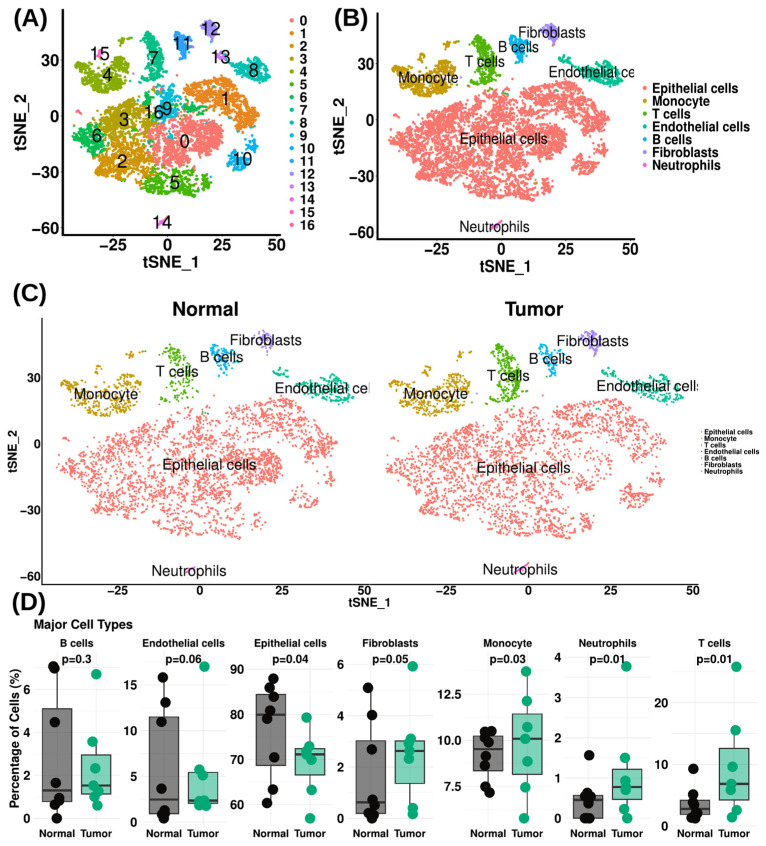
Single-cell transcriptomic profiling reveals altered cellular composition in prostate cancer. (**A**) tSNE representation of the integrated single-cell RNA seq dataset showing clustering of cells from normal and prostate cancer tissues, with 17 transcriptionally distinct clusters. (**B**) Annotation of clusters to seven major cell types using SingleR: epithelial cells (C0, C1, C2, C3, C5, C6, C9, C10, C16), monocytes (C4, C15), T cells (C7), endothelial cells (C8, C13), B cells (C11), fibroblasts (C12), and neutrophils (C14). (**C**) Comparative frequency analysis highlighting shifts in key immune and epithelial populations between normal and prostate cancer tissues, emphasizing changes in epithelial cells, monocytes, and T cells across conditions. (**D**) Quantitative comparison of cell type proportions between normal and prostate cancer samples, illustrating reduced epithelial and endothelial populations alongside increased infiltration of immune cells, including T cells, monocytes, fibroblasts, and neutrophils in tumor tissues.

Numerous cell clusters between normal and prostate cancer were examined by combining the samples, as demonstrated by the tSNE plot (Figure 2A). Furthermore, cells were clustered using the “FindCluster” function to generate 17 clusters (Figure 2A). Following that, we identified seven different cell types inside these clusters using SingleR, a computational annotation program based on the Human Primary Cell Atlas database. These cell types included epithelial cells (cluster annotated: C0, C1, C2, C3, C5, C6, C9, C10 and C16), monocytes (cluster annotated: C4 and C15), T cells (cluster annotated: C7), endothelial cells (cluster annotated: C8 and C13), B cells (cluster annotated: C11), fibroblasts (cluster annotated: C12) and neutrophils (cluster annotated: C14) (Figure 2A, Supplementary Table S1). The frequency analysis revealed significant alterations in epithelial cell, monocyte and T cell populations between normal and prostate tumors. Specifically, epithelial cells exhibited a significant decrease in tumor tissue compared to normal samples. In contrast, T cells showed a significant increase in tumor tissue relative to normal tissue. Moreover, monocytes demonstrated a significant increase in tumor tissue compared with the normal group (Figure 2B). The percentages of each population in the normal and prostate cancer (PCa) samples were subsequently compared, and it was found that within the analyzed cellular populations, the proportion of epithelial cells (PCa vs. normal: 69.33% vs. 76.47%) (*p* = 0.04), B cells (PCa vs. normal: 2.42% vs. 2.82%) (*p* = 0.3), and endothelial cells (PCa vs. normal: 5.16% vs. 5.87%) (*p* = 0.06) was decreased in PCa tissues, whereas the proportion of T cells (PCa vs. normal: 9.65% vs. 3.55%) (*p* = 0.01), monocytes (PCa vs. normal: 9.81% vs. 9.17%) (*p* = 0.03), fibroblasts (PCa vs. normal: 2.50% vs. 1.67%) (*p* = 0.05), and neutrophils (PCa vs. normal: 1.13% vs. 0.46%) (*p* = 0.01) was increased (Figure 2C, Supplementary Table S2). A full list of markers is presented in Supplementary Table S3.

### 3.3. Trajectory Analysis of Cellular Dynamics in Prostate Tumors

**Figure 3:**
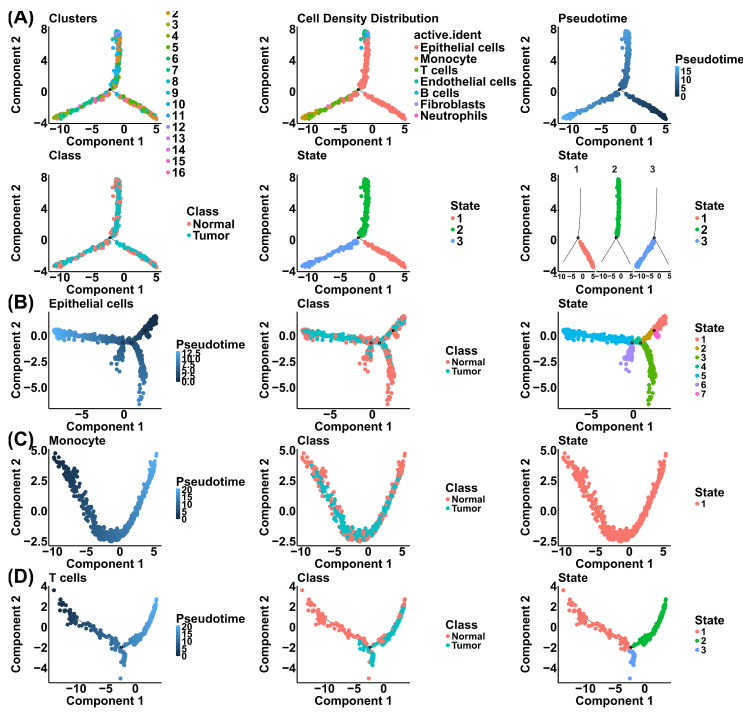
Pseudotime trajectory analysis reveals dynamic lineage remodeling within the prostate tumor microenvironment. (**A**) Overall pseudotime trajectory reconstructed from integrated single-cell RNA-seq data illustrating the hierarchical organization of major epithelial, immune, and stromal populations, with a well-defined topological separation between normal and tumor states. (**B**) Lineage-specific trajectory of epithelial cells showing branching developmental paths and state transitions that distinguish tumor-derived epithelial cells from normal counterparts, consistent with enhanced cellular plasticity during malignant progression. (**C**) Monocyte pseudotime trajectory depicting a highly branched architecture with multiple transitional states, reflecting progressive differentiation and polarization along tumor-associated macrophage-like programs within the tumor microenvironment. (**D**) T cell trajectory analysis depicting state transitions from early to late differentiation stages, suggesting functional remodeling toward terminal differentiation or exhaustion in response to persistent tumor-associated signals.

The single-cell pseudotime trajectory analysis demonstrated the developmental dynamics and lineage relationships of the tumor microenvironment (TME) components compared to normal tissue. The global manifold reconstructs a complex cellular hierarchy comprising diverse populations, including epithelial cells, monocytes, T cells, endothelial cells, B cells, fibroblasts, and neutrophils (Figure 3A). This trajectory reveals a distinct topological separation between “normal” and “tumor” classes, suggesting transcriptional differences associated with the malignant state (Figure 3A). The pseudotime gradient suggests a continuum of differentiation, branching into three primary functional states that may correspond to different lineage commitments or pathological alterations (Figure 3A). To dissect these trajectory patterns, we performed lineage-specific trajectory modeling on the epithelial compartment, revealing a bifurcated structure with three distinct states (Figure 3B). The distribution of cells along this trajectory shows a clear divergence where tumor-derived epithelial cells occupy distinct branches compared to their normal counterparts and suggesting potential lineage plasticity associated with malignant transformation or epithelial-to-mesenchymal transition (EMT) (Figure 3B). The trajectory evaluation of monocytes revealed a highly complex branching architecture with seven distinct states (Figure 3C). This high-dimensional structure may indicate substantial phenotypic plasticity, potentially reflecting the diverse differentiation pathways of monocytes as they are recruited to the tissue and polarize into various tumor-associated macrophage (TAM) phenotypes over pseudotime (Figure 3C). Finally, assessment of T cells follows a trajectory branching into three states (Figure 3D). This progression may reflect transitions of T cells within the TME, potentially mapping the route from naïve or effector states towards terminal differentiation or exhaustion, possibly associated with chronic antigenic exposure in tumor samples (Figure 3D). Collectively, these analyses highlight how the tumor microenvironment is associated with altered developmental trajectories of both malignant and immune cell populations.

### 3.4. Immune Cell Interactions Highlight Key Pathways in Prostate Cancer

**Figure 4:**
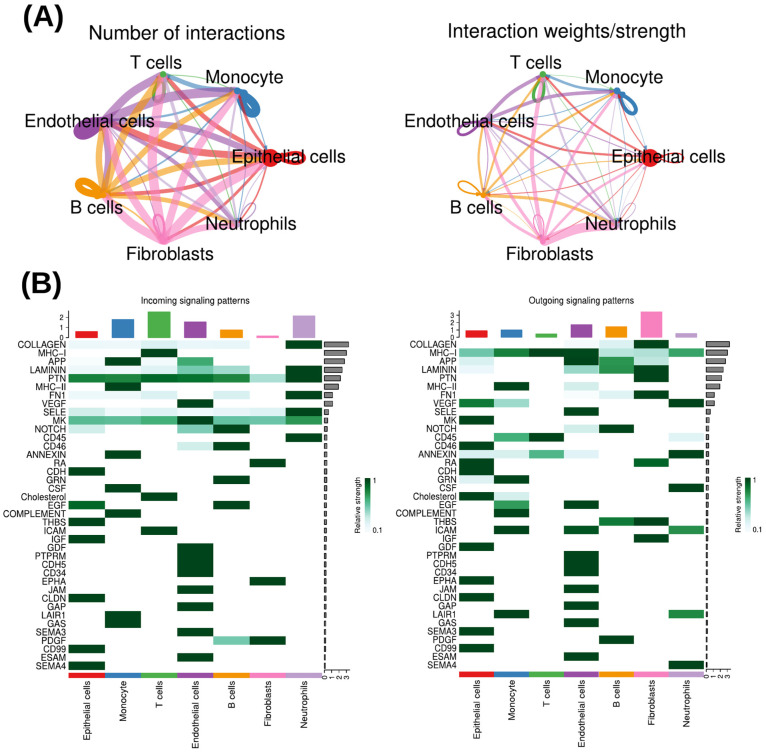
CellChat analysis uncovers cell-type-specific and directional communication networks in the prostate cancer microenvironment. (**A**) Intercellular communication network inferred from single-cell RNA-seq data highlighting epithelial cells, monocyte, fibroblasts, T cells, and endothelial cells as major signaling hubs, based on interaction number and communication strength. (**B**) Comparative analysis of incoming and outgoing ligand-receptor signaling pathways across key cell populations, revealing shared reciprocal signaling programs within individual cell types and directed crosstalk between epithelial cells, monocytes, and T cells that collectively shape immune regulation and tumor-stromal interactions within the tumor microenvironment.

To systematically investigate intercellular communication within the prostate cancer microenvironment, we applied the CellChat framework to infer ligand-receptor-mediated signaling interactions across the single-cell RNA-sequencing dataset. Analysis of the global communication network revealed that epithelial cells, fibroblasts, T cells, and endothelial cells exhibited the highest number of interactions as well as the strongest interaction weights, identifying these populations as major communication hubs within the tumor microenvironment (Figure 4A). Comparative analysis of incoming and outgoing signaling pathways within each cell type revealed distinct patterns of reciprocal communication. In epithelial cells, CD99 (CD99-molecule-mediated signaling), CLDN (claudin-mediated cell-cell junction signaling), and CDH (cadherin-mediated cell adhesion signaling) were consistently identified in both incoming and outgoing interactions, highlighting their central roles in epithelial cell adhesion, junctional organization, and bidirectional signal exchange (Figure 4B). Monocytes displayed shared LAIR1 (leukocyte-associated immunoglobulin-like receptor 1 signaling), COMPLEMENT (complement system signaling), and MHC-II (major histocompatibility complex class II signaling) in both signal reception and transmission, reflecting reciprocal immune-regulatory and antigen-presentation-associated communication patterns (Figure 4B). In T cells, MHC-I (major histocompatibility complex class I signaling) was the only signaling pathway common to both incoming and outgoing interactions, underscoring its dominant role in T-cell-mediated immune surveillance and adaptive immune communication (Figure 4B). Beyond shared pathways, several signaling pathways that were not common between incoming and outgoing interactions within individual cell types were found to participate in intercellular communication with other cell populations. In epithelial cells, incoming EGF (epidermal growth factor signaling pathway) was also observed as outgoing signaling from monocytes, indicating directed epithelial-monocyte communication (Figure 4B). Conversely, epithelial-derived outgoing pathways such as VEGF (vascular endothelial growth factor signaling pathway), EPHA (ephrin type-A receptor signaling), and MK (midkine signaling) were not involved in epithelial signal reception but instead contributed to signaling toward other cell types within the tumor microenvironment (Figure 4B). For monocytes, ICAM (intercellular adhesion molecule signaling), while not shared between incoming and outgoing interactions within monocytes, was detected as incoming signaling in T cells, supporting monocyte-T cell crosstalk (Figure 4B). Similarly, EGF (epidermal growth factor signaling pathway) functioned exclusively as an outgoing pathway in monocytes but acted as an incoming signal in epithelial cells (Figure 4B). Additional monocyte-specific incoming pathways, including CSF (colony-stimulating factor signaling) and GAS (growth-arrest-specific protein signaling), were restricted to monocyte signal reception and were not observed as outgoing pathways in monocytes (Figure 4B). In T cells, non-shared incoming pathways such as ICAM (intercellular adhesion molecule signaling) and Cholesterol (cholesterol-related signaling) were not observed as outgoing signals (Figure 4B). However, ICAM signaling overlapped with outgoing signaling from monocytes, further highlighting immune communication (Figure 4B). Conversely, CD45 (protein tyrosine phosphatase receptor type C signaling), identified as an outgoing pathway in T cells, was also observed in monocyte outgoing signaling, suggesting coordinated activation and regulatory signaling among immune cell populations (Figure 4B).

### 3.5. Single-Cell and Pseudotime Trajectories Uncover TXNIP and BIRC3 as Molecular Signatures in Prostate Cancer Progression

**Figure 5:**
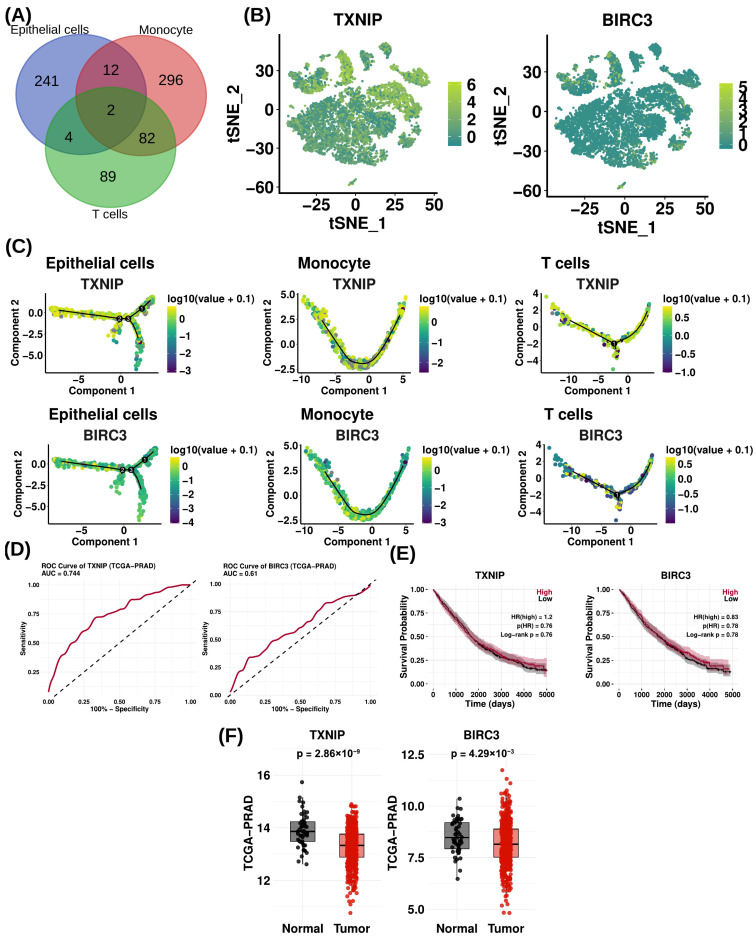
Integrated single-cell and trajectory analyses identify *TXNIP* and *BIRC3* as central molecular regulators during prostate cancer progression. (**A**) Intersection analysis of gene sets derived from epithelial cells, monocytes, and T cells identified as the most influential populations based on integrated single-cell transcriptomics and pseudotime trajectories analyses and reveals *TXNIP* and *BIRC3* as the only genes shared across all three cell types. (**B**) tSNE visualization showing cell-type-specific expression patterns of *TXNIP* and *BIRC3* across prostate cancer samples, with *TXNIP* expressed in epithelial cells, monocytes, and T cells and *BIRC3* predominantly enriched in immune populations, particularly monocytes and T cells. (**C**) Pseudotime trajectory analysis across epithelial cells, monocytes, and T cells illustrating dynamic expression changes in *TXNIP* and *BIRC3* during prostate cancer progression, with progressively increased expression along differentiation trajectories. (**D**) Receiver operating characteristic (ROC) curves based on the TCGA-PRAD cohort demonstrating the diagnostic performance of *TXNIP* and *BIRC3* in distinguishing tumor from normal prostate tissues. (**E**) Kaplan-Meier survival analysis indicating no significant association between *TXNIP* or *BIRC3* expression levels and overall survival in prostate cancer patients. (**F**) Bulk transcriptomic analysis from the TCGA-PRAD cohort showing significant downregulation of *TXNIP* and upregulation of *BIRC3* in prostate tumor tissues compared with normal controls.

To identify candidate molecular features associated with prostate cancer, we focused on three major cell populations in the tumor microenvironment, including epithelial cells, monocytes, and T cells. Candidate genes were initially identified by performing an intersection analysis of gene signatures across these three cell types, which resulted in the selection of *TXNIP* and *BIRC3* (Figure 5A). These genes were subsequently characterized through multiple complementary analyses, including single-cell expression profiling, pseudotime trajectory analysis, biomarker evaluation, survival analysis, and differential expression analysis using TCGA-PRAD datasets, followed by validation in spatial transcriptomics data. In prostate cancer samples, TSNE visualization revealed clear cell-type-specific expression patterns for the candidate genes such as *TXNIP* and *BIRC3* (Figure 5B). *TXNIP* was predominantly expressed in epithelial cells, monocytes, and T cells, whereas *BIRC3* expression was mainly enriched in immune cell populations, particularly T cells and monocytes (Figure 5B). To further investigate the dynamic expression of these genes, pseudotime trajectory analysis was performed across epithelial cells, monocytes, and T cells (Figure 5C). *TXNIP* expression gradually increased along the pseudotime trajectory, showing the highest expression in epithelial cells, followed by monocytes and T cells, suggesting its potential involvement in epithelial state transitions and tumor-immune interactions during prostate cancer progression (Figure 5C). Similarly, *BIRC3* exhibited dynamic expression changes along pseudotime trajectories in all three cell types, with the highest expression observed in epithelial cells, followed by monocytes and T cells, indicating a possible association with later stages of cellular differentiation and immune-related regulatory processes (Figure 5C). The diagnostic performance of *TXNIP* and *BIRC3* in prostate cancer was evaluated using ROC curve analysis based on the TCGA-PRAD dataset. *TXNIP* demonstrated moderate discriminatory ability between tumor and normal prostate tissues (AUC = 0.744), while *BIRC3* showed limited diagnostic performance (AUC = 0.61) (Figure 5D). Survival analysis revealed that the expression levels of *TXNIP* and *BIRC3* were not significantly associated with overall survival in prostate cancer patients (Figure 5E). High *TXNIP* expression was not correlated with poorer survival outcomes (HR = 1.2, log-rank *p* = 0.76), and similarly, elevated *BIRC3* expression showed no significant association with patient survival (HR = 0.83, log-rank *p* = 0.78) (Figure 5E). Finally, analysis of bulk transcriptomic data from the TCGA-PRAD cohort demonstrated distinct expression patterns of the two genes in tumor samples (Figure 5F). *TXNIP* was significantly downregulated in prostate tumor tissues compared to normal controls (*p* = 2.86 × 10^−9^) (Figure 5F), whereas *BIRC3* was significantly upregulated in tumor samples (*p* = 4.29 × 10^−3^) (Figure 5F). Collectively, these findings highlight the cell-type-specific and context-dependent expression patterns of *TXNIP* and *BIRC3* revealed through integrated single-cell and transcriptomic analyses, supporting their consideration as candidate molecular markers associated with tumor–immune interactions in prostate cancer.

### 3.6. Functional Enrichment Analysis of TXNIP and BIRC3 Across Cell Populations in Prostate Cancer

Gene Ontology (GO) and KEGG pathway enrichment analyses were conducted to characterize the functional associations of *TXNIP* and *BIRC3* across distinct cell populations identified in the prostate cancer single-cell RNA-seq dataset. For *TXNIP*, enrichment of biological processes demonstrated pronounced cell-type-specific patterns. In epithelial cells, *TXNIP* was significantly enriched in epidermal cell differentiation (GO:0009913, *p* = 0.004). In monocytes, *TXNIP* was associated with the platelet-derived growth factor receptor signaling pathway (GO:0048008, *p* = 0.03). Notably, in T cells, *TXNIP* showed strong enrichment in the response to tumor cell biological process (GO:0002347, *p* = 0.0001) (Supplementary Table S4). At the molecular function level, *TXNIP* was consistently enriched for enzyme inhibitor activity (GO:0004857) across multiple cell types, including endothelial cells (*p* = 0.001), epithelial cells (*p* = 0.0003), monocytes (*p* = 0.0003), and T cells (*p* = 0.02) (Supplementary Table S4). No significant enrichment was observed for cellular component categories. KEGG pathway analysis revealed that, in T cells, *TXNIP* was significantly associated with immune-related pathways, including the T cell receptor signaling pathway (hsa04660, *p* = 7.74 × 10^−10^) and natural killer cell-mediated cytotoxicity (hsa04650, *p* = 1.55 × 10^−9^) (Supplementary Table S4). For *BIRC3*, biological process enrichment was predominantly related to inflammatory and tumor necrosis factor-associated signaling. In epithelial cells, *BIRC3* was enriched in response to tumor necrosis factor (GO:0034612, *p* = 0.01) and cellular response to tumor necrosis factor (GO:0071356, *p* = 0.03). In monocytes, highly significant enrichment was observed for regulation of inflammatory response (GO:0050727, *p* = 3.95 × 10^−21^), positive regulation of innate immune response (GO:0045089, *p* = 2.69 × 10^−18^), the innate immune response-activating signaling pathway (GO:0002758, *p* = 2.69 × 10^−16^), canonical NF-kappaB signal transduction (GO:0007249, *p* = 3.00 × 10^−9^), and the tumor necrosis factor-mediated signaling pathway (GO:0033209, *p* = 0.001). Consistent inflammatory signatures were also observed in other immune populations. In neutrophils, *BIRC3* was enriched in regulation of inflammatory response (GO:0050727, *p* = 1.22 × 10^−5^). In T cells, enrichment was detected for regulation of inflammatory response (GO:0050727, *p* = 5.21 × 10^−6^), positive regulation of innate immune response (GO:0045089, *p* = 9.65 × 10^−6^), and cellular response to tumor necrosis factor (GO:0071356, *p* = 0.002) (Supplementary Table S4). No significant enrichment was identified for molecular function, cellular component, or KEGG pathways for *BIRC3*.

### 3.7. Spatially Resolved Cellular Landscapes and TXNIP-BIRC3 Expression Across Progressive States of Human Prostate Cancer

**Figure 6:**
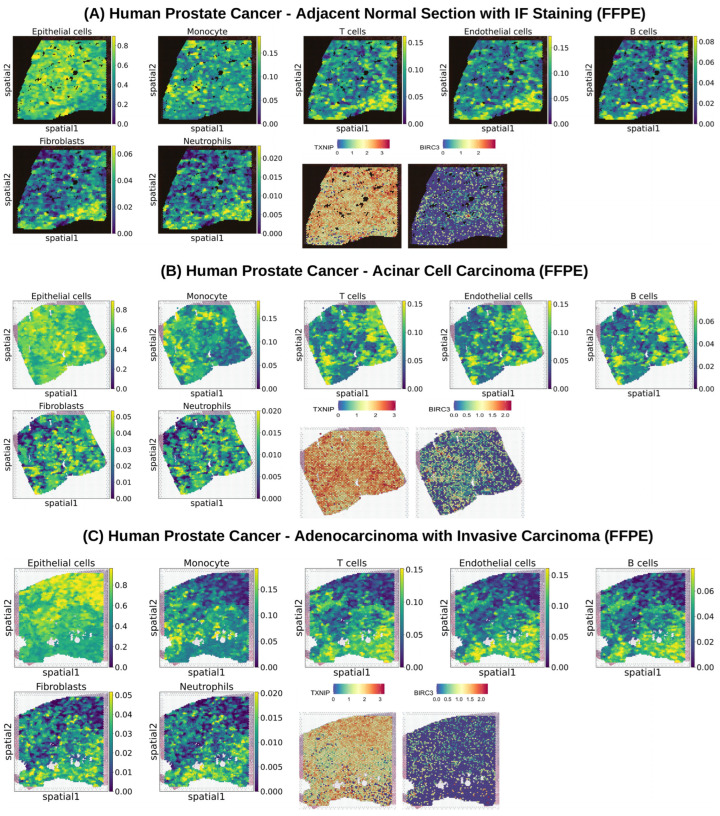
Spatial transcriptomics reveals tissue-state-specific cellular organization and regional expression patterns of *TXNIP* and *BIRC3* in prostate cancer. (**A**) Spatial mapping of major epithelial, immune, and stromal cell populations in adjacent normal prostate tissue (FFPE), showing epithelial cells as the dominant population localized primarily in central regions, with monocytes showing moderate abundance and immune cell subsets displaying intermediate, region-specific distributions. Spatial gene expression analysis demonstrates widespread *TXNIP* expression and minimal *BIRC3* expression, indicating predominant *TXNIP*-associated transcriptional activity in adjacent normal tissue. (**B**) Spatial organization of cell populations in acinar cell carcinoma (FFPE) tissue, with epithelial cells strongly enriched in central tumor regions and increased representation of fibroblasts and neutrophils compared to adjacent normal tissue. *TXNIP* exhibits high spatial expression across large tumor areas, while *BIRC3* shows moderate regional expression, suggesting enhanced metabolic or stress response programs alongside intermediate inflammatory signaling in acinar carcinoma. (**C**) Spatial distribution of cellular populations in invasive adenocarcinoma (FFPE) tissue, revealing a more heterogeneous immune-stromal microenvironment with widespread infiltration of immune and stromal cells. *TXNIP* expression remains elevated across invasive tumor regions, whereas *BIRC3* expression is relatively limited, indicating persistent *TXNIP*-associated transcriptional programs despite advanced tumor invasion.

We performed spatial transcriptomics analysis using 10x Genomics standard FFPE datasets on three prostate cancer tissue sections, including Human Prostate Cancer, Adjacent Normal Section with IF Staining (FFPE), Human Prostate Cancer, Acinar Cell Carcinoma (FFPE), and Human Prostate Cancer, Adenocarcinoma with Invasive Carcinoma (FFPE), to characterize the spatial distribution of major epithelial, immune, and stromal cell populations within the intact tissue architecture and to evaluate the regional expression patterns of *TXNIP* and *BIRC3* across spatially defined regions of the tissue sections (Figure 6A–C). In the adjacent normal prostate tissue, epithelial cells represented the most abundant cell population and were predominantly localized within the central regions of the tissue (Figure 6A). Monocytes showed moderate abundance and were mainly distributed across the central and upper-left regions, whereas T cells, endothelial cells, and B cells displayed intermediate abundance with a preferential localization in the central and upper-right regions (Figure 6A). In contrast, fibroblasts and neutrophils were observed at low abundance and were sparsely localized to the upper-right areas of the tissue section, indicating limited stromal and neutrophil infiltration in adjacent normal regions (Figure 6A). This spatial mapping allowed for the visualization of regional cellular heterogeneity and the organization of immune and stromal populations relative to epithelial compartments. Spatial gene expression analysis revealed high expression of *TXNIP* across substantial regions of the tissue, whereas *BIRC3* expression remained low, suggesting higher *TXNIP*-associated transcriptional activity in adjacent normal prostate tissue and highlighting region-specific variation in gene expression across spatial spots (Figure 6A). In acinar cell carcinoma tissue, epithelial cells were again the most dominant population, with strong enrichment in the central regions of the tumor (Figure 6B). Monocytes, T cells, endothelial cells, and B cells were present at moderate levels, with monocytes mainly localized to the central and upper-left regions and the remaining immune and vascular populations enriched in the central and upper-right regions (Figure 6B). Notably, fibroblasts and neutrophils exhibited a moderate abundance in this tissue type and were primarily distributed within the central tumor regions, indicating increased stromal and innate immune involvement compared to adjacent normal tissue (Figure 6B). At the transcriptional level, *TXNIP* exhibited very high spatial expression throughout large areas of the tumor, whereas *BIRC3* showed moderate expression, suggesting potentially enhanced *TXNIP*-related metabolic or stress response activity alongside an intermediate association with *BIRC3*-related inflammatory pathways in acinar carcinoma and reflecting spatially heterogeneous transcriptional programs across tumor regions (Figure 6B). In adenocarcinoma tissue with invasive features, epithelial cells remained the most abundant population and were predominantly localized in the central and upper regions of the tissue section (Figure 6C). Monocytes showed moderate abundance with enrichment in the central and upper-left regions, while T cells, endothelial cells, B cells, fibroblasts, and neutrophils were moderately abundant and distributed mainly across the central and lower regions of the invasive tumor area, reflecting a more heterogeneous immune-stromal tumor microenvironment with distinct spatial organization of cellular populations (Figure 6C). Spatial expression mapping demonstrated high *TXNIP* expression across the tissue, whereas *BIRC3* expression was relatively low, indicating that *TXNIP*-associated transcriptional programs may remain prominent even in invasive prostate cancer regions, while *BIRC3*-associated activity appears more limited, demonstrating how spatial transcriptomics enables the simultaneous evaluation of gene expression patterns and local cellular composition across tumor regions (Figure 6C).

### 3.8. Spatial Co-Localization of TXNIP and BIRC3 with Tumor-Associated Cell Types in Prostate Cancer Microenvironments

**Figure 7:**
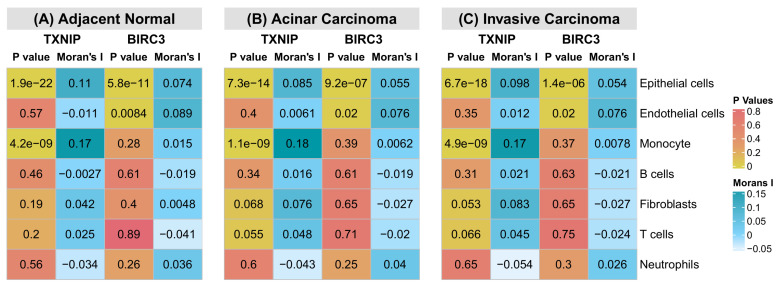
Spatial co-localization analysis reveals reproducible alignment of *TXNIP* and *BIRC3* expression with tumor-associated cell populations during prostate cancer progression. (**A**) Moran’s-I-based spatial co-occurrence analysis in adjacent normal prostate tissue (FFPE) demonstrating significant spatial association of *TXNIP* with epithelial cells and monocytes and *BIRC3* with epithelial and endothelial cells, indicating coordinated gene expression within cell-type-enriched regions. (**B**) Spatial co-localization patterns in acinar cell carcinoma (FFPE) tissue showing persistent association of *TXNIP* with epithelial and monocyte-rich regions and significant alignment of *BIRC3* expression with epithelial structures and vascular-associated regions. (**C**) Spatial co-occurrence analysis in invasive prostate adenocarcinoma (FFPE) tissue revealing sustained association of *TXNIP* with epithelial and monocyte regions, alongside selective enrichment of *BIRC3* expression in epithelial and endothelial compartments within invasive tumor areas. Across all tissue states, *TXNIP* displays consistent epithelial-myeloid spatial co-localization, whereas *BIRC3* maintains epithelial association, with endothelial co-localization emerging specifically in malignant contexts.

To investigate whether local gene expression aligns with cell type abundance in adjacent normal prostate tissue, we examined spatial co-occurrence patterns using Moran’s I statistics. In the Human Prostate Cancer, Adjacent Normal Section with IF Staining (FFPE) dataset, *TXNIP* demonstrated a strong spatial association with epithelial cells (*p* = 1.95 × 10^−22^) and monocytes (*p* = 4.19 × 10^−9^), indicating that regions enriched for these cell types exhibited coordinated *TXNIP* expression (Figure 7A). In addition, *BIRC3* showed a significant spatial association with epithelial cells (*p* = 5.78 × 10^−11^) and endothelial cells (*p* = 0.008), suggesting localized *BIRC3* expression within epithelial- and endothelial-enriched regions of adjacent normal prostate tissue (Figure 7A). No other significant spatial associations were observed between gene expression and cell type distributions in this tissue. Spatial co-localization analysis in Human Prostate Cancer, Acinar Cell Carcinoma (FFPE) tissue revealed significant associations between gene expression and specific cell populations. *TXNIP* exhibited significant spatial association with epithelial cells (*p* = 7.33 × 10^−14^) and monocytes (*p* = 1.12 × 10^−9^), indicating that *TXNIP* expression was preferentially localized to epithelial- and monocyte-rich regions within acinar carcinoma tissue (Figure 7B). Furthermore, *BIRC3* showed a significant spatial association with epithelial cells (*p* = 9.23 × 10^−7^) and endothelial cells (*p* = 0.01), suggesting coordinated expression of *BIRC3* in regions characterized by epithelial structures and vascular components (Figure 7B). No additional significant gene–cell type associations were detected. In Human Prostate Cancer, Adenocarcinoma with Invasive Carcinoma (FFPE) tissue, Moran’s I analysis demonstrated significant spatial co-localization between gene expression and distinct cell populations. *TXNIP* was significantly associated with epithelial cells (*p* = 6.70 × 10^−18^) and monocytes (*p* = 4.88 × 10^−9^), indicating localized enrichment of *TXNIP* expression within epithelial- and monocyte-dense invasive tumor regions (Figure 7C). Additionally, *BIRC3* exhibited significant spatial association with epithelial cells (*p* = 1.36 × 10^−6^) and endothelial cells (*p* = 0.02), suggesting that *BIRC3* expression is selectively enriched in epithelial- and vasculature-associated regions of invasive prostate adenocarcinoma (Figure 7C). Across all three prostate tissue types examined, *TXNIP* consistently exhibited significant spatial association with epithelial cells and monocytes, indicating a reproducible alignment of *TXNIP* expression with epithelial- and myeloid-enriched regions irrespective of tissue state (Figure 7A–C). In parallel, *BIRC3* showed a consistent spatial association with epithelial cells across all samples, while its association with endothelial cells emerged specifically in malignant tissues (Figure 7A–C). These shared patterns suggest that epithelial-cell-associated transcriptional programs represent a common spatial feature of *TXNIP* and *BIRC3* expression across prostate cancer progression.

## 4. Discussion

The molecular landscape of prostate cancer (PCa) is characterized by a complicated interaction between malignant epithelial cells and a dynamic tumor microenvironment (TME) [42]. Despite recent advances in genomic profiling, it is still highly challenging to discover the spatiotemporal architecture and intercellular communication networks that accelerate the spread of PCa [43]. In this study, we integrated single-cell RNA sequencing (scRNA-seq) with spatial transcriptomics data to construct a high-resolution atlas of the prostate TME. Our multi-omics approach discovered particular lineage trajectories associated with tumor development, identified considerable remodeling of cellular compositions, and spatially indicated the interaction network with an emphasis on the important regulatory genes such as *BIRC3* and *TXNIP*. The single-cell transcriptome analysis demonstrated that prostate cancer significantly changes the prostatic cellular composition, with noticeable modifications in the tissue and stromal compartment. The complication of normal secretory structure and the possible deformation or loss of normal luminal phenotype that contribute to PCa progression could be the causes of the observed considerable decrease in epithelial cells [44]. A significant increase in T lymphocytes, monocytes, and neutrophils, as well as increases in fibroblasts, on the other hand, demonstrates enhanced inflammatory infiltration and a reactive tumor microenvironment [45]. This widespread infiltration of myeloid and lymphocyte lineages indicates that the tumor creates a chronic inflammatory niche, where long-term immune activation may unexpectedly promote stromal remodeling and tumor survival instead of effective anti-tumor immunity [46]. Our pseudotime trajectory analyses highlighted the phenotypic plasticity associated with PCa progression [47]. The topological separation of normal and tumor epithelial cells into distinct trajectory branches suggests transcriptional reprogramming associated with malignant transformation, potentially reflecting the acquisition of invasive properties or an epithelial-to-mesenchymal transition (EMT) [48]. This lineage divergence has been reported as a characteristic feature of aggressive malignancies, allowing tumor cells to adapt to metabolic stress and survive therapeutic pressure [48]. Similarly, the immune compartment exhibited complex developmental dynamics [49]. Monocytes displayed a highly branched architecture with seven distinct states, suggesting a spectrum of differentiation states as they are recruited from the periphery and polarized within the TME [50]. This adaptability supports evidence for the concept that monocytes are dynamically formed by components derived from tumors rather than remaining as a set of identical cells [51]. Additionally, the T cell progression, which split into three stages, may show how naïve phenotypes transition to effector function and, finally, terminal differentiation or failure to function [52]. A crucial aspect of the tumor-immune interface is this developmental process, where long-term exposure to antigens causes T cells to become dysfunctional, which makes immune evasion easier [53,54]. The survival of prostate cancer cells may also be influenced by supportive signaling from the stromal and immune compartments [46]. Our CellChat analysis identified epithelial cells, fibroblasts, and immune subsets as central communication hubs. Notably, we observed a directional signaling axis where monocytes provide epidermal growth factor (EGF) signals to epithelial cells. This observation suggests a potential paracrine interaction in which myeloid cells may influence epithelial cell survival and proliferation [55]. Conversely, epithelial cells interact with the larger TME through VEGF and Midkine (MK) signaling, which could lead to angiogenesis and tissue remodeling [56,57]. Furthermore, the immune crosstalk specifically identified the interaction between monocytes and T cells via ICAM and MHC pathways, which underscores the complexity of antigen presentation and immune regulation in the TME [58]. While MHC-I and MHC-II pathways were active, potentially indicating ongoing immune surveillance, the simultaneous engagement of adhesion and regulatory pathways suggests a delicate balance between immune activation and suppression [59]. The identification of *TXNIP* (thioredoxin-interacting protein) and *BIRC3* (baculoviral IAP repeat containing 3) as crucial molecular indicators that connect the epithelial and immunological compartments was an essential finding of our integrated analysis. In our findings, bulk transcriptomic analysis showed that *TXNIP* was downregulated in tumors, which is consistent with its canonical function as a negative regulator of glucose uptake [60], and single-cell data showed that it was highly expressed in certain immune and epithelial subpopulations within the tumor tissue. The progressive increase in *TXNIP* along the pseudotime trajectory suggests that it may be induced as a stress response mechanism in surviving tumor cells or infiltrating immune cells facing metabolic strain, such as hypoxia or glucose deprivation [61,62]. Its spatial co-localization with monocytes further suggests a potential association with metabolic coordination between the tumor and myeloid cells [63]. In contrast, *BIRC3*, an inhibitor of apoptosis, showed upregulation in bulk tumor samples and was enriched in immune populations in our single-cell data. Functionally, *BIRC3* was enriched in NF-κB and TNF signaling pathways [64]. By inhibiting apoptosis in the presence of inflammatory signals, *BIRC3* may contribute to the survival of tumor cells and tumor-infiltrating lymphocytes within an inflamed microenvironment [65]. The integration of spatial transcriptomics provided critical architectural context to our single-cell findings, providing specific insights about the targeted therapy of prostate cancer. In adjacent normal tissues, the structured localization of *TXNIP* within epithelial regions is consistent with a spatially organized metabolic state, whereas the transition to acinar cell carcinoma was accompanied by a more heterogeneous spatial distribution of *TXNIP*, suggesting a potential alteration in this architecture [10]. In acinar carcinoma, the co-localization of *TXNIP* and *BIRC3* with epithelial and monocyte-rich regions suggests the presence of localized microenvironmental niches potentially associated with cell survival [66]. From a clinical perspective, this suggests that treatments that target metabolic pathways associated with *TXNIP* could be more effective when combined with immunomodulatory therapies that prevent monocyte recruitment in these particular niches [67]. Furthermore, our analysis of adenocarcinoma with invasive carcinoma tissue revealed a more disorganized and spatially heterogeneous distribution of stromal and immune elements. A key finding in these invasive regions was the specific spatial association of *BIRC3* with endothelial cells, a feature not observed in adjacent normal tissue. While this spatial pattern indicates a correlation rather than a causal relationship, it raises the possibility that in invasive PCa tissue, *BIRC3* may be associated with mechanisms related to pathological angiogenesis, potentially through interactions that could influence endothelial cell survival within the inflammatory TME [68]. Consequently, targeting *BIRC3* could offer a dual therapeutic benefit such as sensitizing tumor cells to apoptosis and simultaneously normalizing the tumor vasculature to improve drug delivery [69]. While the spatial data demonstrates that the TME is not homogeneous, it is crucial to fully understand these regional differences, particularly the immune-stromal interactions in invasive regions compared to the tumor core, with the goal of developing spatially suitable therapeutic approaches. This study has several limitations that should be considered when interpreting the findings. First, the analyses were conducted using publicly available datasets, including standard Visium FFPE spatial transcriptomics data. Reliance on public datasets may introduce dataset-specific biases related to sample selection, sequencing depth, and preprocessing strategies. In addition, the relatively small number of available single-cell and spatial transcriptomic samples limits the ability to fully capture patient-level heterogeneity in prostate cancer, and re-analysis in larger, independent cohorts will be necessary to improve robustness and generalizability. Second, the intrinsic resolution constraints of the standard 10x Genomics Visium FFPE platform (55 µm capture spots) should be considered. Each spatial spot may capture transcripts originating from multiple neighboring cells, which limits single-cell resolution and may affect the interpretation of fine-scale spatial relationships, particularly for vascular structures and localized microenvironmental niches. Because each Visium FFPE capture spot aggregates transcripts from multiple adjacent cells, the observed endothelial enrichment and its correlation with *BIRC3* expression represent spot-level spatial associations rather than true single-cell colocalization. Mixed epithelial-stromal composition within nearby spots may partially contribute to the endothelial signal, and therefore these spatial relationships should be interpreted with appropriate caution. Third, our findings are primarily computational and require experimental validation at both gene and protein expression levels. The lack of spatial metabolomics data also prevents the direct assessment of metabolite distributions and metabolic pathway activity within spatial tissue contexts. Future studies should incorporate larger patient cohorts, higher-resolution spatial technologies, spatial metabolomics approaches, and mechanistic experiments in experimental models, together with validation in human clinical samples, to further confirm the biological and translational relevance of these findings in prostate cancer.

## 5. Conclusions

Our research performed an integrated multi-omics approach to dissect the cellular and spatial heterogeneity of the prostate tumor microenvironment. We discovered a complex signaling network where myeloid cells can actively promote tumor growth by highlighting the developmental paths of immune suppression and epithelial adaptability. Our spatial transcriptomics discoveries demonstrate that *TXNIP* and *BIRC3* are not only differentially expressed genes but also regionally established regulators of inflammatory survival and metabolic stress. The distinct spatial co-localization of *BIRC3* with the tumor vasculature in invasive carcinoma tissue highlights a novel, potentially druggable interaction. These findings provide a high-resolution molecular atlas that uncovers specific vulnerabilities within the tumor-immune interactome, suggesting that targeting the spatial niches defined by *TXNIP* and *BIRC3* expression could represent an effective approach for precision intervention in prostate cancer.

## Data Availability

Publicly available datasets were analyzed in this study. The single-cell RNA-Seq dataset can be found at GSE176031 (https://www.ncbi.nlm.nih.gov/geo/query/acc.cgi?acc=GSE176031, accessed on 27 March 2026) from Gene Expression Omnibus (GEO, https://www.ncbi.nlm.nih.gov/geo/) and spatial transcriptomics datasets, including Human Prostate Cancer, Adjacent Normal Section with IF Staining (FFPE) [https://www.10xgenomics.com/datasets/human-prostate-cancer-adjacent-normal-section-with-if-staining-ffpe-1-standard]; aggressive Stage IV Human Prostate Cancer, Acinar Cell Carcinoma (FFPE) [https://www.10xgenomics.com/datasets/human-prostate-cancer-acinar-cell-carcinoma-ffpe-1-standard]; and the Stage III Human Prostate Cancer, Adenocarcinoma with Invasive Carcinoma (FFPE) [https://www.10xgenomics.com/datasets/human-prostate-cancer-adenocarcinoma-with-invasive-carcinoma-ffpe-1-standard-1-3-0], can be downloaded from https://www.10xgenomics.com/datasets. All other data supporting the findings of this study are available within the article and the supporting information or from the corresponding author upon reasonable request.

## Supplementary Materials

The following supporting information can be downloaded at: https://www.mdpi.com/article/10.3390/cells15070647/s1, Figure S1: Quality control and principal component evaluation of scRNA-seq data from prostate cancer samples; Table S1: Cluster annotation for cell types; Table S2: Percentage for cell types; Table S3: Markers in normal-tumor group; Table S4: Full list of pathways (biological process, molecular function, cellular component and KEGG Pathway Enrichment Analysis) for each cell type in prostate cancer.

## Code Availability

Original codes were developed for preprocessing of single-cell RNA-Seq and spatial transcriptomics data, and all codes are available from the lead contact upon request. Other data analysis approaches, including the algorithm and packages, are described in the Materials and Methods section.

## Abbreviations

The following abbreviations are used in this manuscript:

TXNIP	Thioredoxin-Interacting Protein
BIRC3	Baculoviral IAP Repeat Containing 3
scRNA-seq	Single-Cell RNA Sequencing
ST	Spatial Transcriptomics
FFPE	Formalin-Fixed Paraffin-Embedded
PCa	Prostate Cancer
BRCA1	Breast Cancer Type 1 Susceptibility Protein
BRCA2	Breast Cancer Type 2 Susceptibility Protein
PTEN	Phosphatase and Tensin Homolog
TP53	Tumor Protein p53
TME	Tumor Microenvironment
IF Staining	Immunofluorescence Staining
SRA	Sequence Read Archive
GEO	Gene Expression Omnibus
QC	Quality Control
CCA	Canonical Correlation Analysis
PCA	Principal Component Analysis
t-SNE	t-Distributed Stochastic Neighbor Embedding
GO	Gene Ontology
KEGG	Kyoto Encyclopedia of Genes and Genomes
RDS	R Data Serialization File
RCTD	Robust Cell Type Decomposition
ROC	Receiver Operating Characteristic
TCGA-PRAD	The Cancer Genome Atlas Prostate Adenocarcinoma
AUC	Area Under the Curve
FDR	False Discovery Rate
C	Cell Cluster
EMT	Epithelial–Mesenchymal Transition
TAM	Tumor-Associated Macrophage
CD99	Cluster of Differentiation 99
CLDN	Claudin Family Proteins
CDH	Cadherin Family Proteins
LAIR1	Leukocyte-Associated Immunoglobulin-Like Receptor 1
COMPLEMENT	Complement System
MHC-II	Major Histocompatibility Complex Class II
MHC-I	Major Histocompatibility Complex Class I
EGF	Epidermal Growth Factor
VEGF	Vascular Endothelial Growth Factor
EPHA	Ephrin Type-A Receptor
MK	Midkine
ICAM	Intercellular Adhesion Molecule
CSF	Colony-Stimulating Factor
GAS	Growth-Arrest-Specific Genes
CD45	Protein Tyrosine Phosphatase Receptor Type C
