# Supplementary material for: Single-Cell and Spatial Transcriptomics Reveal That TXNIP and BIRC3 Contribute to Human Prostate Tumor Progression
Source: Cells. 2026 Apr 2;15(7):647. doi: 10.3390/cells15070647 (PMC13072731; doi:10.3390/cells15070647)

Single-Cell and Spatial Transcriptomics Reveal TXNIP and BIRC3 Contribute to Human Prostate Tumor Progression

Seyed Taleb Hosseini [1,2], Hossein Azizi [3,*] and Thomas Skutella [4,*]

Supplementary Figures

Supplementary Figure S1. Quality control and principal component evaluation of scRNA-seq data from prostate cancer samples. (A) Quality control filtering of scRNA-seq data illustrating the removal of low-quality cells prior to downstream analysis. **(B)** Identification of the top 2,000 highly variable genes selected for dimensionality reduction and clustering analyses. **(C)** Principal component analysis (PCA) performed on highly variable genes using the RunPCA function to capture major sources of transcriptional variability across samples. **(D)** JackStrawPlot showing statistically significant principal components with low p-values and their associated variance contributions. **(E)** ElbowPlot depicting the variance explained by successive principal components and guiding the selection of an appropriate dimensionality cutoff. **(F)** Heatmap of the leading principal components highlighting genes with the highest contributions to each component, illustrating distinct transcriptional drivers across PCs. **(G)** Visualization of gene loadings across principal components using VizDimLoadings, supporting the identification of biologically informative PCs for downstream tSNE analysis.

(A) Normal samples – Before Quality control

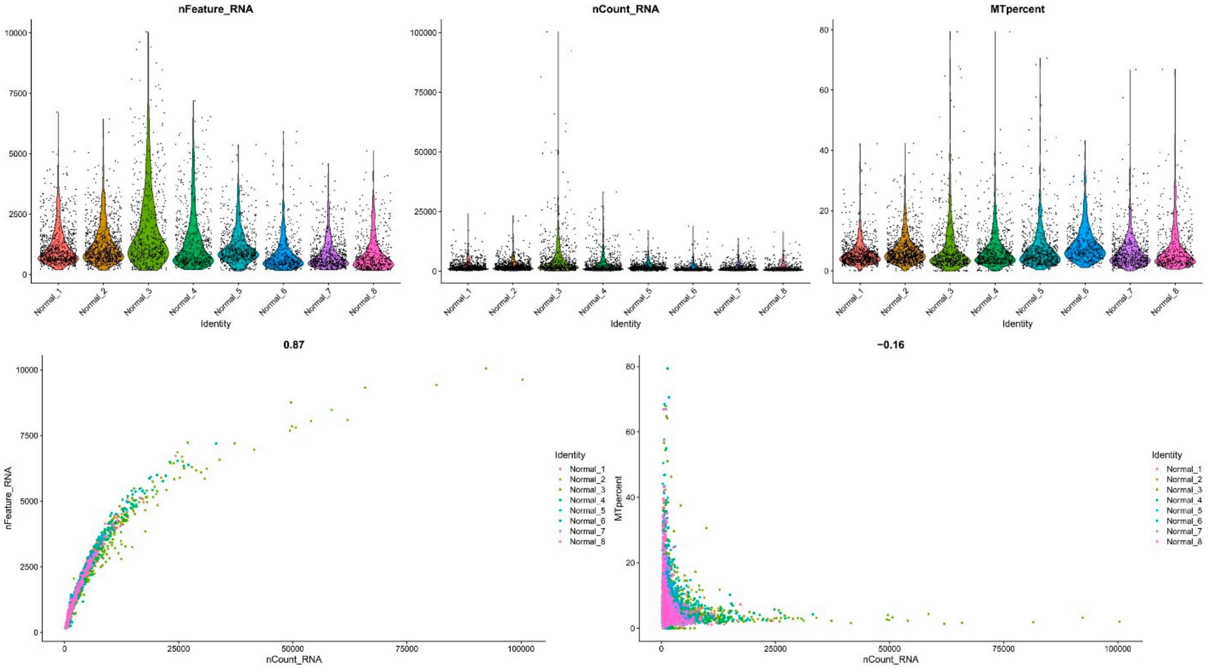

(A) Normal samples – After Quality control

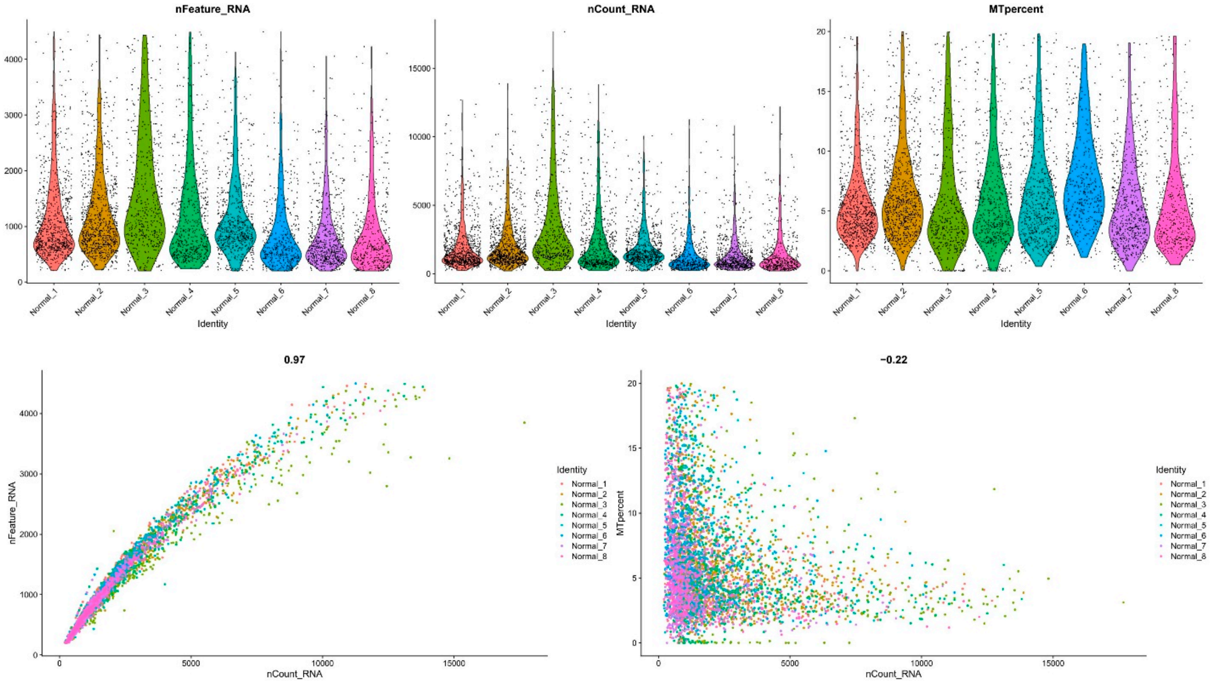

(A) Tumor samples – Before Quality control

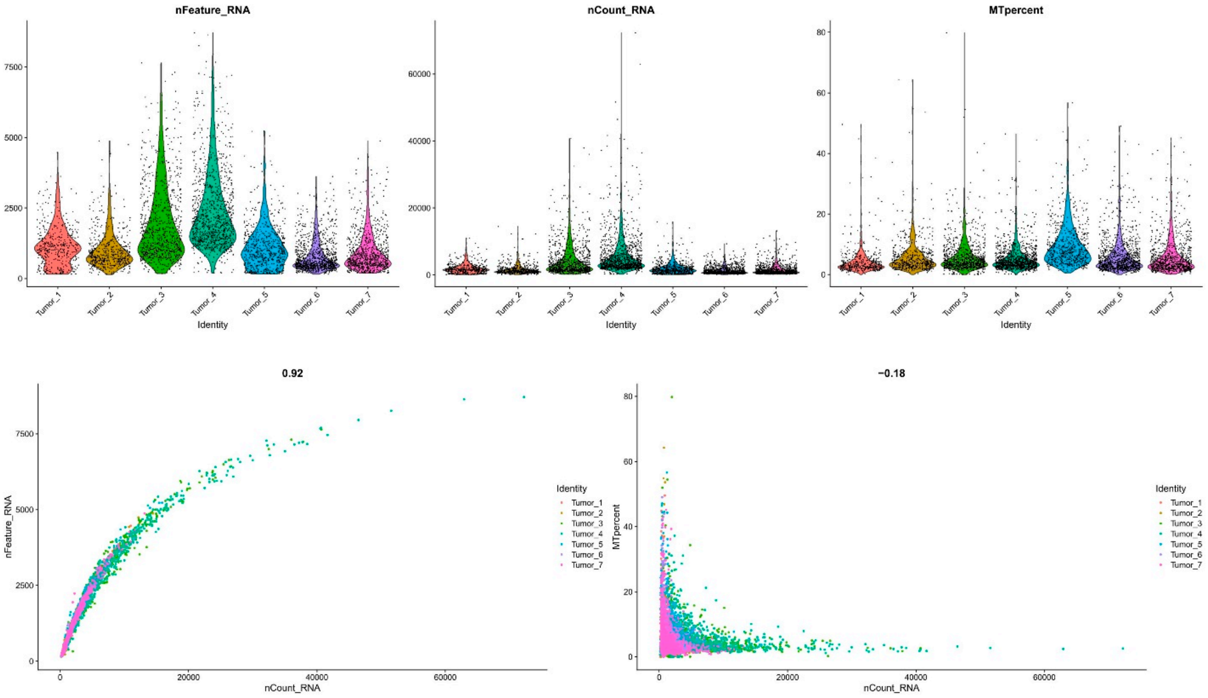

(A) Tumor samples – After Quality control

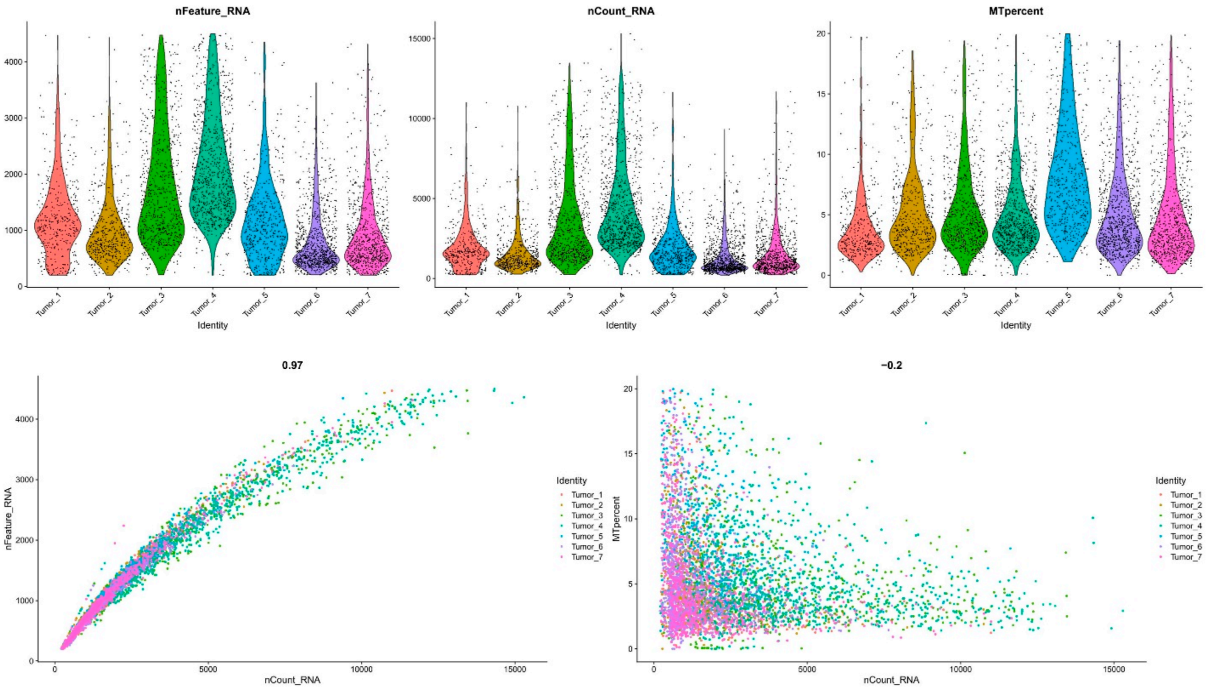

(B) Normal samples

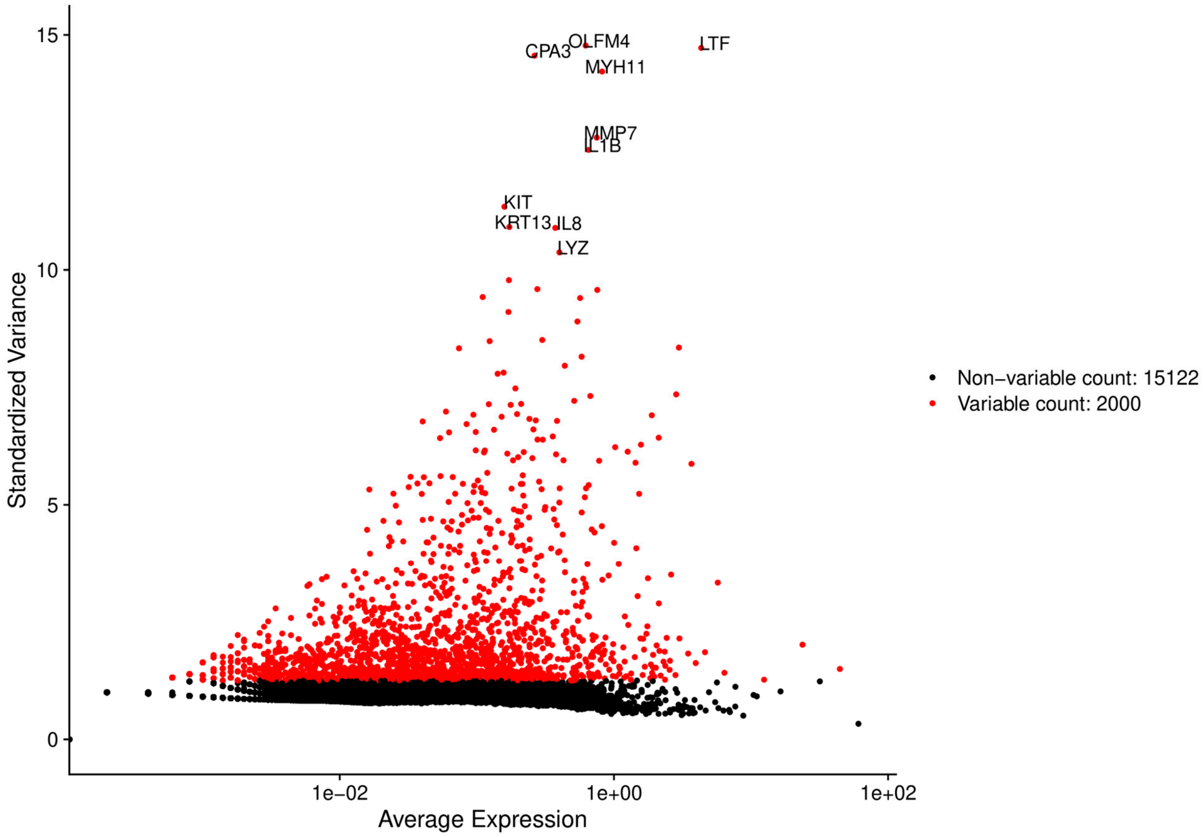

(B) Tumor samples

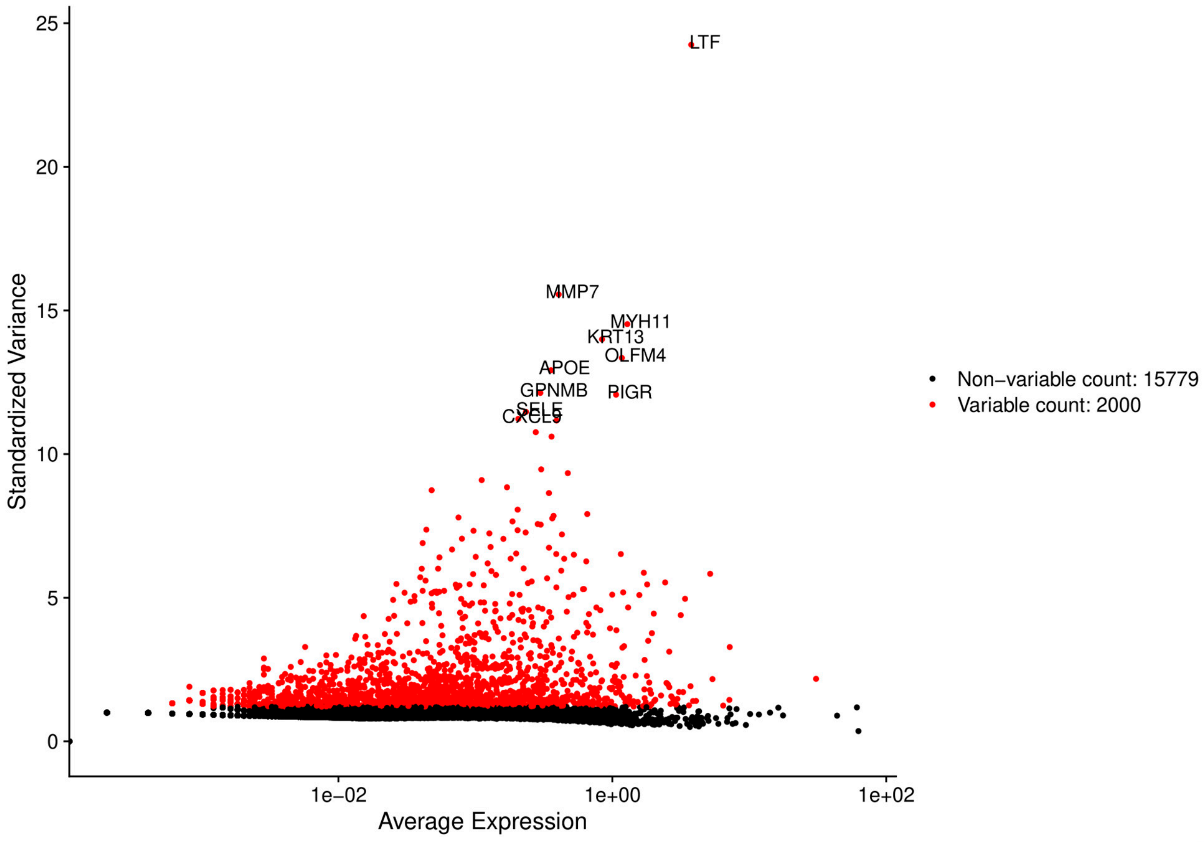

(C) Principal component analysis (PCA) - DimPlot

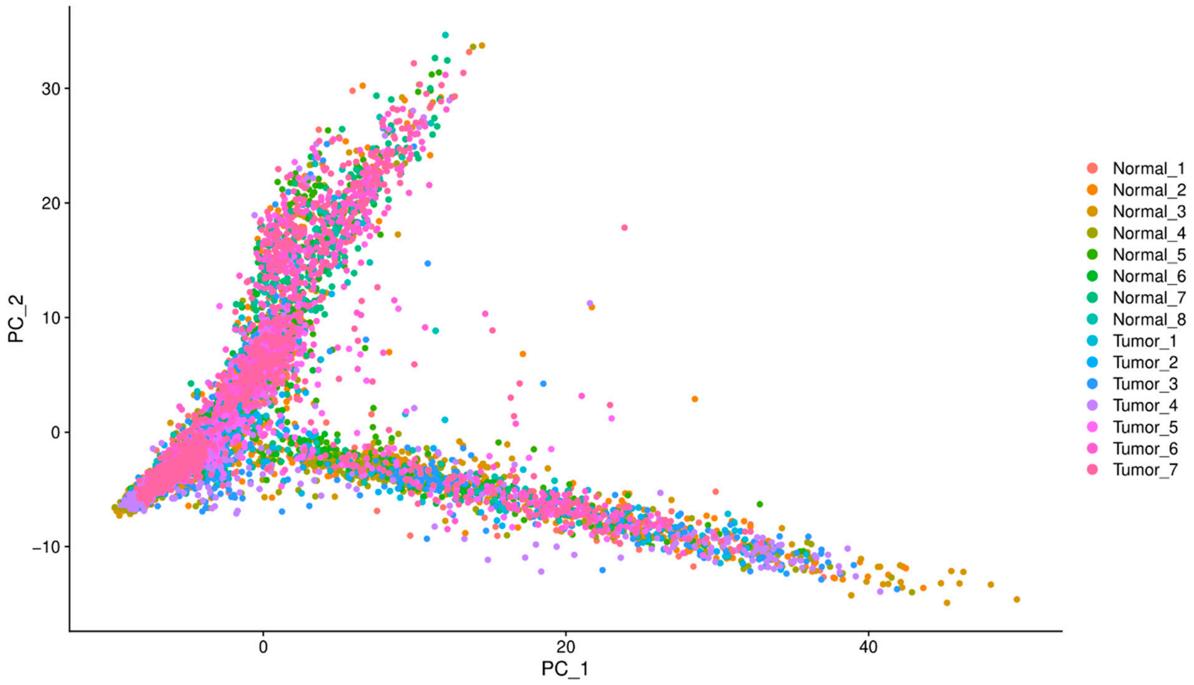

(D) JackStrawPlot

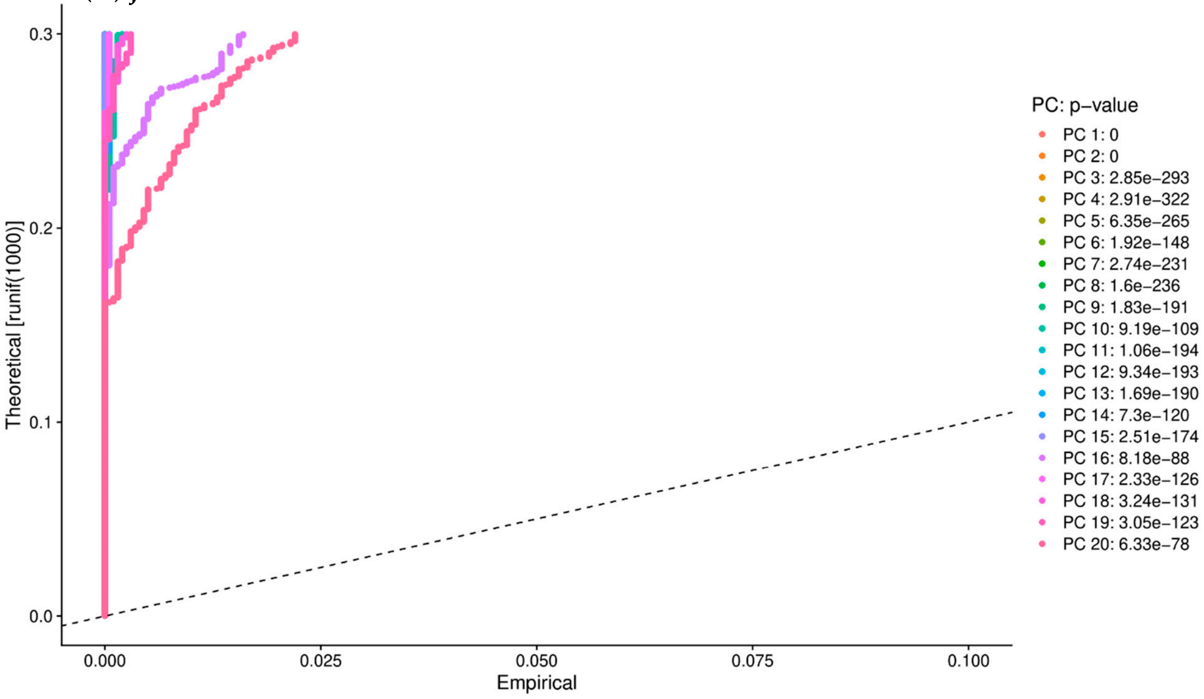

(E) ElbowPlot

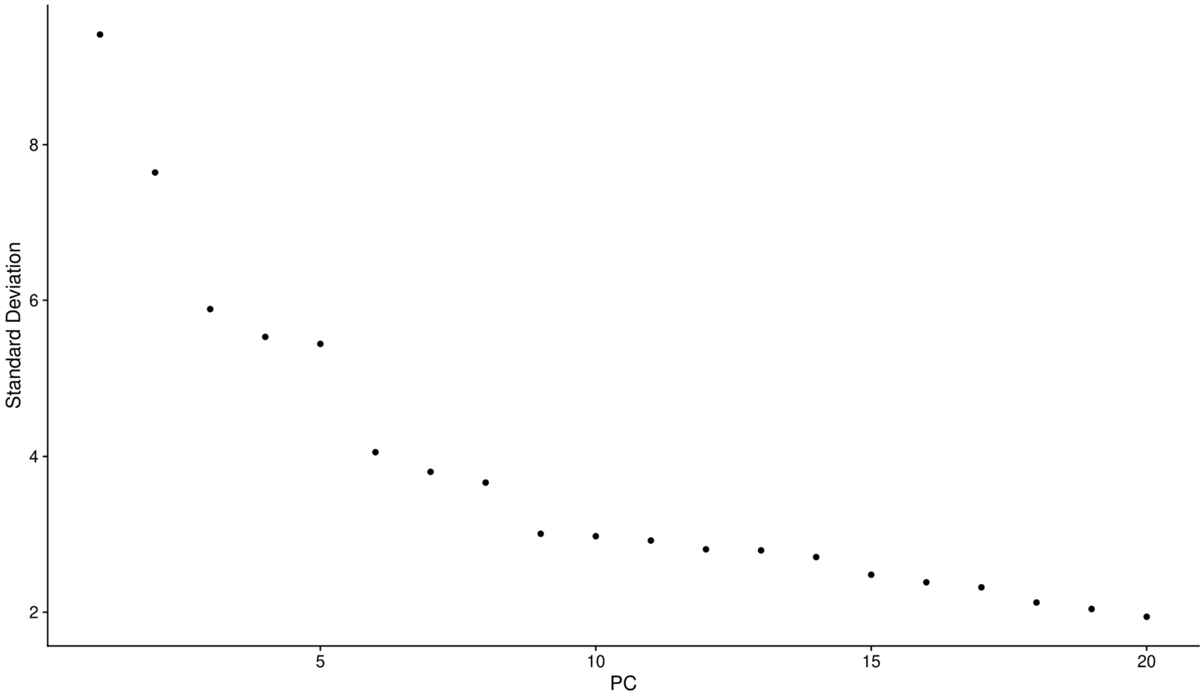

(F) DimHeatmap

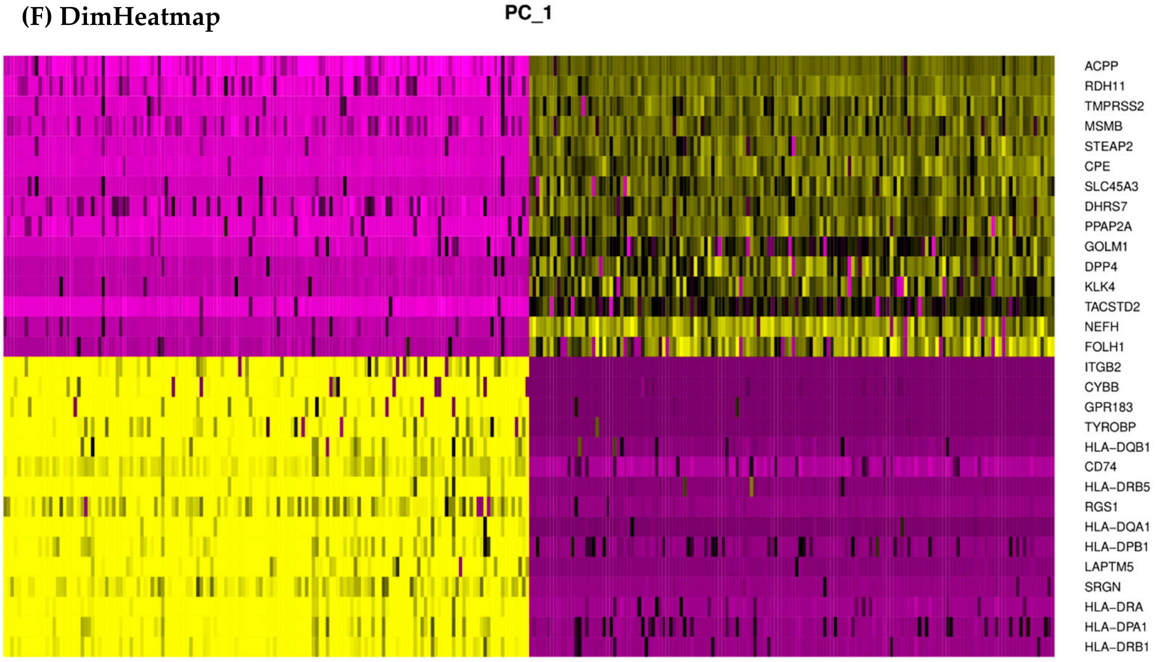

(G) VizDimLoadings

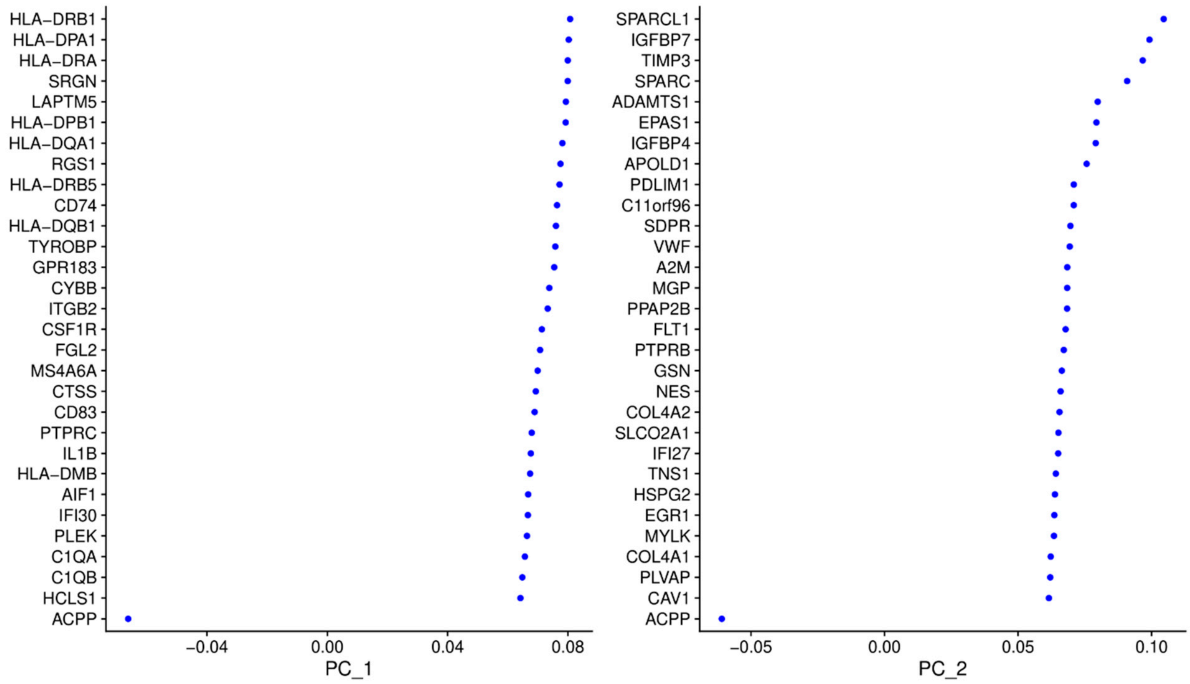

Supplement: Supplementary file 1 [file cells-15-00647-s001.zip › Supplementary Figures.pdf]
